# Microfluidic Nanoparticle Separation for Precision Medicine

**DOI:** 10.1002/advs.202411278

**Published:** 2024-12-04

**Authors:** Zhenwei Lan, Rui Chen, Da Zou, Chun‐Xia Zhao

**Affiliations:** ^1^ School of Chemical Engineering, Faculty of Sciences, Engineering and Technology The University of Adelaide Adelaide SA 5005 Australia

**Keywords:** microfluidic, nanomedicine, nanoparticles, precision medicine, separation

## Abstract

A deeper understanding of disease heterogeneity highlights the urgent need for precision medicine. Microfluidics, with its unique advantages, such as high adjustability, diverse material selection, low cost, high processing efficiency, and minimal sample requirements, presents an ideal platform for precision medicine applications. As nanoparticles, both of biological origin and for therapeutic purposes, become increasingly important in precision medicine, microfluidic nanoparticle separation proves particularly advantageous for handling valuable samples in personalized medicine. This technology not only enhances detection, diagnosis, monitoring, and treatment accuracy, but also reduces invasiveness in medical procedures. This review summarizes the fundamentals of microfluidic nanoparticle separation techniques for precision medicine, starting with an examination of nanoparticle properties essential for separation and the core principles that guide various microfluidic methods. It then explores passive, active, and hybrid separation techniques, detailing their principles, structures, and applications. Furthermore, the review highlights their contributions to advancements in liquid biopsy and nanomedicine. Finally, it addresses existing challenges and envisions future development spurred by emerging technologies such as advanced materials science, 3D printing, and artificial intelligence. These interdisciplinary collaborations are anticipated to propel the platformization of microfluidic separation techniques, significantly expanding their potential in precision medicine.

## Graphical abstract

This review provides a comprehensive review of microfluidic nanoparticle separation for precision medicine, covering the fundamentals of passive, active, and hybrid separation techniques. It focuses on recent advances in their application to liquid biopsy and nanomedicine. Additionally, the review discusses advancements in 3D printing, artificial intelligence, and advanced materials that support the practical translation of these techniques.

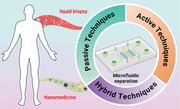

## 1 Introduction

Precision medicine, also known as personalized medicine, represents a modern approach to healthcare that considers individual genetic, environmental, and lifestyle differences. Unlike the traditional one‐size‐fits‐all approach, precision medicine customizes treatments based on each patient's unique biological makeup and personal circumstances.^[^ ^1^ ^]^ The concept of precision medicine has long been fundamental to medical practice, guiding efforts to classify diseases and tailor treatments based on specific diagnoses. What is novel today is the rapid pace of advancements in diagnostic and therapeutic options.^[^ ^2^ ^]^ The integration of genetics, informatics, and imaging, along with innovations in cell sorting, proteomics, epigenetics, and metabolomics, is significantly broadening the capability of precision medicine.^[^ ^3^, ^4^, ^5^ ^]^

**Figure 1:**
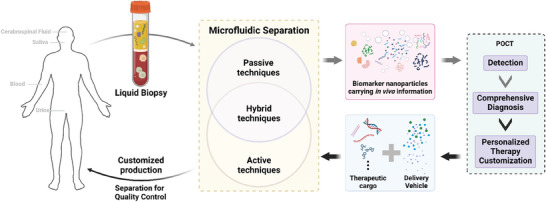
The role of microfluidic nanoparticle separation in precision medicine. Microfluidic technologies, including active, passive, and hybrid techniques, enable the effective isolation of key biomarkers from patient‐derived biological fluids. In parallel, point‐of‐care testing (POCT) supports rapid detection and diagnosis, guiding the design of tailored treatment strategies. In producing personalized therapy, microfluidic techniques are essential for controlling nanoparticle synthesis and purification, thereby tuning their properties such as purity, particle size, and drug loading.

Liquid biopsy provides a minimally invasive method for diagnostic and prognostic applications.^[^ ^6^, ^7^ ^]^ It allows for the analysis of readily accessible biological fluids, such as blood, urine, and saliva fluid, facilitating dynamic disease monitoring.^[^ ^8^ ^]^ Bioactive nanoparticles, including secretory proteins and extracellular vesicles (EVs, including exosomes), provide clinicians with sensitive and sustainable insights into disease progression.^[^ ^6^, ^9^ ^]^ Therefore, separation of these bioactive nanoparticles from complex body fluids can effectively enhance the accuracy of diagnosis and analysis (**Figure** **1**). For example, effective separation of EVs can remove contaminants such as microvesicles, proteins, nucleic acids, thereby achieving higher detection accuracy.^[^ ^10^, ^11^, ^12^ ^]^

On the other side, nanomedicine is also an important part of precision medicine by leveraging nanoscale materials and technologies to diagnose, treat and monitor diseases with improved precision.^[^ ^13^, ^14^, ^15^ ^]^ Nanoparticles can be engineered to detect biomarkers at very low concentrations,^[^ ^16^, ^17^, ^18^ ^]^ and can also be designed for various therapeutic delivery applications (e.g., small molecular drugs, RNAs, etc.).^[^ ^19^, ^20^, ^21^, ^22^ ^]^ The synthesis and purification of these nanoparticles are crucial for ensuring their safety, efficacy, and reproducibility in clinical applications.^[^ ^23^, ^24^, ^25^ ^]^

**Figure 2:**
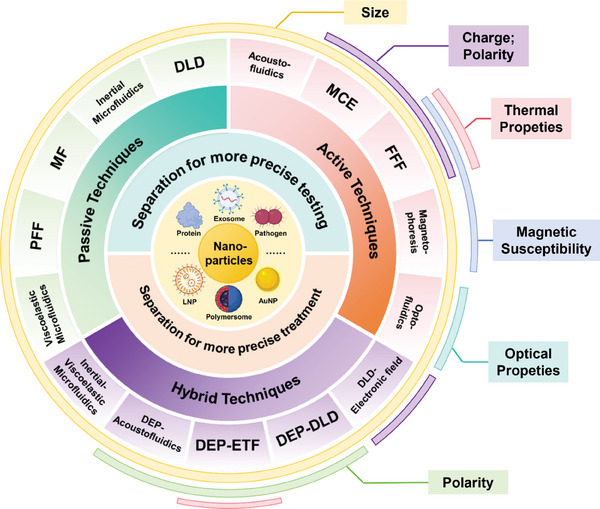
Microfluidic separation techniques for nanoparticles. Passive techniques include viscoelastic microfluidic, pinched flow fractionation (PFF), microfluidic filtration (MF), inertial microfluidic, and deterministic lateral displacement (DLD). Active techniques include acoustic fluidics, microfluidic chip electrophoresis (MCE), field flow fractionation (FFF), magnetophoresis, and optofluidics. Hybrid techniques mainly include the combination of inertial microfluidic and viscoelastic microfluidic (inertial‐ viscoelastic microfluidic), Dielectrophoresis and acoustofluidics (DEP‐ acoustofluidics), DEP and electrothermal fluid (DEP‐ETF), DEP and DLD (DEP‐DLD), and DLD with electronic field (DLD‐ electronic field). The colour label in the outer ring indicates the corresponding particle properties commonly exploited by these microfluidic separation techniques.

Traditional methods, such as ultrafiltration, ultracentrifugation, and size exclusion chromatography, rely on particle size, often resulting in substantial sample loss and potential particle damage.^[^ ^26^, ^27^, ^28^ ^]^ In the current precision medicine landscape, preserving the integrity of the biological information carried by nanoparticles, along with other properties, such as charge, shape, and mechanical characteristics, has become essential.^[^ ^29^, ^30^ ^]^ The advent of microfluidic technology has opened vast possibilities for these nanoparticles’ separation, contributing to personalized diagnosis of disease and facilitating the clinical translation of nanomedicine in precision medicine. Initially, particle separation on microfluidic platforms relied on intricate structural designs using fluid dynamics and channel geometries, termed passive techniques.^[^ ^31^ ^]^ Subsequently, separation designs mediated by physical fields emerged, known as active techniques. Recently, the integration of various techniques, known as hybrid techniques, has been reported as an approach to enhance separation capacity for nanoscale products in heterogeneous sample mixtures while also reducing potential particle damage during the separation process (**Figure** **2**).^[^ ^32^ ^]^

Microfluidic technology naturally meets the demands of precision medicine for accurate, rapid, and efficient processing of small‐volume, high‐value fluid samples.^[^ ^33^ ^]^ It offers significant potential benefits in disease detection, monitoring, and personalized treatment.^[^ ^5^, ^34^, ^35^ ^]^ The precise control, high‐throughput capabilities, modularity, and minimal sample requirements of microfluidic platforms make them well suited for translating research findings into practical applications in precision medicine.^[^ ^36^, ^37^ ^]^ In diagnostics, microfluidics can facilitate the rapid detection and analysis of nanoparticulate biomarkers, leading to more personalized and timely medical interventions,^[^ ^38^, ^39^ ^]^ Moreover, they can be used for efficient and accurate sorting of nanoparticles for targeted drug delivery systems, enhancing the specificity and efficacy of treatments.^[^ ^40^, ^41^ ^]^ While microfluidic‐based separation technologies have shown promising potential in lab research, their success in clinics has been limited, especially at the nanoscale and with bioactive soft particles. Limitations include restricted manipulation of nanoparticles, potential damage to biological particles due to energy transfer, and low adaptability to different fluid properties.^[^ ^42^, ^43^, ^44^, ^45^ ^]^ Moreover, despite the continuous development and expanding applications of microfluidic separation techniques, their widespread adoption in standard laboratories is still limited. This is due to the diverse physical principles underlying various microfluidic separation technologies, which require specific expertise and specialized equipment for fabrication, often exceeding the capabilities of potential users in the biomedical field.

These challenges have prompted researchers and manufacturers to explore new technologies as alternative solutions. Excitingly, advances and intersections in multiple fields are changing the landscape of the microfluidic field for nanoparticle separation applications. Emerging 3D printing technologies such as stereolithography (SL) and 2‐photon polymerization (2PP), has achieved higher manufacturing resolution (tens of microns).^[^ ^46^, ^47^ ^]^ In addition, corresponding advances in materials science that can be used for these high‐resolution 3D printing technologies are also essential, such as PEGDA 258 resins,^[^ ^48^ ^]^ methacrylate‐based PDMS photoresins,^[^ ^49^ ^]^ and *Liquid PMMA*.^[^ ^50^ ^]^ In addition to engineering, data‐driven artificial intelligence (AI) is also challenging traditional microfluidic chip design.^[^ ^51^ ^]^ Effective nanoparticle separation depends on the translation of physical principles, and integrating AI can enhance traceability in design and reduce data requirements by embedding known physical information. For example, Lashkaripour et al. established an “end‐to‐end” automated design platform,^[^ ^52^ ^]^ bringing user needs with domain expertise.

This review focuses on the fundamental principles of microfluidic separation and its application to nanoscale particle separation (less than 1000 nm), highlighting the practical impact on precision medicine from detection to monitoring, and treatment. It guides readers from foundational concepts to quantitative research insights, helping establish a cohesive framework. Finally, it discusses the challenges limiting broader application, such as gaps in manufacturing, equipment, and expertise, while exploring interdisciplinary solutions emerging from material science, advanced manufacturing, and AI‐driven approaches.

## 2 Principles of Particle Separation

The choice of nanoparticle separation methods depends on the purpose and specific properties of the target particles, including size, charge, polarity, shape, and surface characteristics, all of which significantly impact particle function.^[^ ^53^ ^]^ As shown in Figure 2, different microfluidic separation techniques rely on different particle properties.

### 2.1 Size

Particle size is a fundamental characteristic for particle separation, primarily due to its intrinsic physical and chemical properties such as density and reaction activity, and the size‐associated biological performance. This characteristic is particularly crucial when particles are used as drug‐delivery vehicles. Factors such as excretion, blood circulation, and interaction with phagocytes significantly influence their in vivo stability. Generally, a longer systemic circulation time increases the likelihood of particles effectively entering cells to exert their therapeutic effects. However, size‐related considerations are critical. Nanoparticles smaller than 10 nm are typically rapidly cleared by the kidneys, while those larger than 200 nm may activate the complement system, leading to unpredictable outcomes.^[^ ^30^, ^54^ ^]^

Particle size is also considered to be an important characteristic of extracellular vesicle (EVs, ranging in diameter from 30 nm to 10 µm).^[^ ^55^, ^56^ ^]^ More specifically, it can be subdivided into three main categories according to their size: exosomes (30 to 120 nm), microvesicles (150 to 1000 nm), and apoptotic bodies (100 to 5000 nm), suggested by the International Society for Extracellular Vesicles (ISEV) for EV identification.^[^ ^57^, ^58^ ^]^ For metal nanoparticles, such as gold (Au), platinum (Pt), silver (Ag), and palladium (Pd), different particle sizes exhibit different optical characteristics and properties. These properties also change with the length‐to‐diameter ratio of the particles, the thickness of the nano shell, and the concentration. These variations make metal nanoparticles particularly useful in bio‐imaging fields.^[^ ^59^ ^]^

### 2.2 Shape

Although small particles are often modelled as spherical for simplifying force analysis, in reality they exhibit a diverse range of geometric and irregular shapes. The significance of particle shape in various applications is increasingly recognized as more critical than previously understood.^[^ ^60^, ^61^ ^]^ Particles of identical composition and similar size but different shape can display vastly different behaviors due to variations in surface binding capabilities, cellular uptake and release, and optical and plasmonic effects.^[^ ^62^, ^63^, ^64^, ^65^ ^]^ Mitragotri et al.^[^ ^66^, ^67^, ^68^ ^]^ has highlighted these differences, and demonstrated that worm‐like particles inhibit macrophage phagocytosis at cellular level. Furthermore, rod‐shaped particles have been shown higher uptake by breast cancer cells and more accumulation in the brain and lung. For noble metal nanoparticles, their optical properties are intricately linked to their shape, even when the size remains constant. This underscores the crucial of shape as a parameter in the design and application of nanoparticles, particularly in fields like targeted drug delivery and bio‐imaging.^[^ ^69^ ^]^

### 2.3 Charge

At solid‐liquid interfaces, active factors such as dangling bonds or the attachment of charged molecules result in an overall charge on the nanoparticle surface. The charge of nanoparticles influences their behavior, especially their tendency to aggregate, as electrostatic repulsion between them ensures colloidal stability.^[^ ^70^, ^71^ ^]^ This highlights the importance of zeta potential ζ in maintaining the stability of particle solution systems. Additionally, the charges of particles can significantly affect their biodistribution in vivo. A recent research highlight is the selective organ‐targeting (SORT) strategy, where the surface charge of lipid nanoparticles (LNPs) is fine‐tuned by adding complementary SORT molecules. The biodistribution study demonstrated a selective enrichment of SORT LNPs in the lungs, spleen, or liver, which is conducive to targeted therapy and the avoidance of systemic toxicity.^[^ ^72^, ^73^ ^]^ Moreover, upon introduction into biological systems, charged particles can interact with oppositely charged biomacromolecules, affecting their surface properties and biodistribution. For example, apolipoproteins are absorbed onto cationic liposomes in blood vessels to mediate liver targeting. This protein crown structure also affects the bio‐interfacial properties of the nanocarriers, changing the way by which the liposomes cross the membrane from a non‐energy‐dependent membrane fusion mechanism to an energy‐dependent endocytosis mechanism.^[^ ^74^ ^]^

### 2.4 Other Particle Properties

A distinctive feature of particles’ biochemistry is their specificity, characterized by the strong interaction between antigens and antibodies. For example, different subpopulations of EVs possess distinct surface proteins, serving as important markers for their accurate identification and function.^[^ ^75^, ^76^ ^]^ For other nanoparticles that do not have biochemical properties themselves, it is often possible to attach functional units to the particle depending on the intended function and purpose. For example, some nanoparticle‐based delivery strategies achieve stronger targeted delivery by modifying the particle surface with specific proteins.^[^ ^77^, ^78^, ^79^ ^]^ In a study by Rurik et al., CD5 was modified onto the LNP surface to deliver mRNA to T cells that were difficult to deliver in vivo.^[^ ^80^ ^]^ This modification successfully realized in‐situ CAR‐T (Chimeric antigen receptor T cells) engineering, effectively alleviating the process of cardiac injury. In addition to pure targeting function, there are studies that not only achieve targeting, but also enable immunomodulation, such as tumor‐associated macrophage reprogramming.^[^ ^81^, ^82^, ^83^ ^]^ Furthermore, while properties like magnetism, polarity, inertia, compressibility, and thermodynamic properties, are rarely reported to have significant biological applications, understanding them is important for recognizing the differences between target nanoparticles, allowing for the exploitation of these properties for a range of particle manipulation.^[^ ^69^, ^84^, ^85^, ^86^ ^]^

## 3 Microfluidic Techniques for Nanoparticle Separation

The study of smaller particles has spurred a demand for nano‐scale processing of these precious samples. Microfluidics, a technology that emerged in the early 1990s, has been widely used in various fields of nanoparticles, including catalysis of metal nanoparticles^[^ ^87^ ^]^ and synthesis of lipid/polymer nanoparticles,^[^ ^35^, ^88^, ^89^, ^90^ ^]^ as well as their purification, separation and enrichment.^[^ ^91^, ^92^ ^]^ Compared to those traditional methods, microfluidics offers several advantages. Microfluidics provides precise control over small volumes, minimizing the loss of precious samples, while its precise pumping system ensures more repeatable results.^[^ ^93^ ^]^ Moreover, microfluidic devices can be designed as modular systems to accommodate a variety of continuous production devices, allowing different functions to be implemented in series. Additionally, they can also be designed with different structures to meet specific needs, offering unparalleled flexibility.^[^ ^94^, ^95^, ^96^ ^]^ Furthermore, by paralleling the same microfluidic chip or device module, processing throughput can be easily adjusted to meet the desired scale of production.^[^ ^97^, ^98^, ^99^, ^100^ ^]^ This section will first start with the basics of microfluidics, and then discuss the three different microfluidic methods for nanoparticle separation including passive, active and hybrid methods.

### 3.1 Basics of Microfluidics

Turbulence often occurs in large‐scale fluids, resulting in unpredictability within the fluid. Microfluidics, however, can maintain a laminar flow, ensuring control over analysis, observation, and manipulation. This can be explained through quantified parameters, such as the Reynolds number (*Re*), which represents the relationship between viscous and inertial forces. *Re* is defined as below in Equation (1):

(1) $$Re=\frac{\rho vd}{\mu }\;$$

where the ρ is fluid density (kg/m^3^), *v* is the flow speed (m ^−1^s), *d* is the characteristic length (m), and μ is the dynamic viscosity of the fluid (Pa·s or N·s/m^2^). In most cases, fluids are usually water or organic solvents, which have a lower dynamic viscosity. Taking water as an example, flow rates in microfluidics are usually small and microchannel widths are usually in the range of microns, so *Re* is normally less than 2100 for water flowing through microchannels, indicating laminar flow.^[^ ^101^ ^]^

The diffusion of a particle in a microchannel can be described using the Péclet number (*P_e_*), the ratio of convection to diffusion:^[^ ^102^ ^]^

(2) $$P_{e}=\frac{vL}{D_{f}}\;$$

where *v* is the fluid speed, *L* is the channel width, and *D_f_* is the particle diffusion coefficient. *D_f_* is dependent on the particle shape and size. Taking spherical particles as an example, *D_f_* is defined in Equation (3):^[^ ^103^ ^]^

(3) $$D_{f}=\frac{kT}{6\pi \mu a}\;$$

where *k* is the Boltzmann constant, *T* is the absolute temperature, μ is the dynamics fluid viscosity, and *a* is the particle hydrodynamic radius. It is generally believed that when *P_e_* >> 1, the behaviour of particles following fluid movement is much greater than diffusion behaviour, so under the same conditions, the smaller the particles, the larger the *D_f_* will be, resulting in the reduction of *P_e_*.

### 3.2 Passive Microfluidic Techniques

Passive microfluidic techniques separate particles by designing intricate microfluidic structures for controlling fluid interactions, and they have demonstrated advantages in separating soft particles, especially biological particles.^[^ ^104^ ^]^ Five methods have been reported, including deterministic lateral displacement (DLD),^[^ ^105^, ^106^, ^107^ ^]^ microfluidic filtration (MF),^[^ ^108^, ^109^ ^]^ inertial microfluidics,^[^ ^110^, ^111^, ^112^ ^]^ pinched flow fractionation (PFF)^[^ ^113^, ^114^, ^115^ ^]^ and viscoelastic microfluidics,^[^ ^37^, ^116^, ^117^ ^]^ for nanoparticle separation.

#### 3.2.1 Deterministic Lateral Displacement

**Figure 3:**
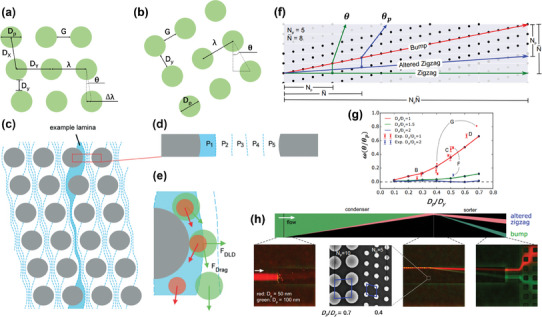
Principle and representative examples of DLD‐based microfluidic separation techniques. a,b) Crucial parameters in two types of DLD structure unit. c,d) The deflection angle (***θ***) of successive pillars and displacement between each row of pillars form the periodicity (N). In a DLD device with *N* = 5, a flow layer within a unit formed 5 streamlines between adjacent pillars (P_1_–P_5_) due to lateral row movement. Adapted with permission.^[^ ^121^ ^]^ Copyright 2009 Royal Society of Chemistry. e) Motion of particles from P_1_ streamline in DLD species. Red particles smaller than ***D***_***c***_ are affected by F_Drag_ and remain in P_1_, while green particles larger than ***D***_***c***_ will move to the next streamline after passing through each cell. Reproduced with permission^[^ ^121^ ^]^ Copyright 2009, Royal Society of Chemistry. f) The migration patterns of a particle's trajectory in a DLD array and the respective angles. g) The correlation between normalized migration angle and the pillar‐to‐pitch ratio in both simulations and experiments. h) An efficient separation structure composed of a condenser module and a sorter module in series, which was used to separate the 50 nm (red) and 100 nm (green) nanoparticles. f–h) were adapted with permission.^[^ ^106^ ^]^ Copyright 2017, National Academy of Sciences.

Deterministic lateral displacement (DLD) separates particles of different sizes by building an array of tilted pillar in a microfluidic channel to generate laminar flow with multiple branches between the pillars. Since Huang et al.^[^ ^118^ ^]^ first reported the DLD technique in 2004, it has been mainly applied to biomedicine related particle separation or analysis, especially for smaller nanoparticles (>20 nm).^[^ ^105^ ^]^ In general, two designs of arrays, rectangles and diamonds, are considered as the DLD unit (**Figure** **3**a,b).

The critical diameter (*D*_c_) plays a key role in determining the separation capability of the designed DLD chip, which is closely related to the number of streamlines of laminar flow between the pillar gaps. Particles larger than *D*_c_ will continuously transfer from the first streamline to the next, thus maintaining a straight path, while particles smaller than *D*_c_ will always follow the initial flow line, forming a zigzag path (Figure 3c,e). *D*_c_ is closely related to the displacement between each row of pillars, and the deflection angle (θ) between them determines the number of streamlines between adjacent pillars and the size of array unit, which was first discovered by Huang et al.^[^ ^118^ ^]^ as periodicity (N). It can be observed that the number of streamlines is the same to the number of cycles, so it could be described as Equation (4):

(4) $$N=\frac{\lambda }{\Delta \lambda }=\frac{1}{tan\theta }=\frac{1}{\varepsilon }\;$$

Inglis et al.^[^ ^119^ ^]^ described *D_c_* as twice as wide as the first streamline (P_1_, shown in Figure 3d). However, Davis et al.^[^ ^120^ ^]^ tested the experimental critical values in DLD devices with different displacement fractions and gap sizes, giving the following formula:

(5) $$D_{c}=1.4G\varepsilon^{0.48}$$

The key parameters affecting *D_c_* are the deflection Angle of the array and the gap of adjacent pillars. This equation is implemented based on *D_x_*/ *D_y_* =  1. Kim et al.^[^ ^106^ ^]^ demonstrated that the lateral displacement of particles does not always strictly follow the theoretical path, they sometimes do not follow the zigzag path flow in an array unit, so called pseudo period. The effect of the separation error on the separation degree can be expressed by the N_P_ and $\bar{N}$ shown in the Figure 3f. Therefore, the ratio of the modified zigzag mode migration angle to the pillar structural angle under small angles can be described as:

(6) $$\omega =\frac{\theta }{\theta_{p}}\;∼\frac{\bar{N}-N_{P}}{\;\bar{N}}$$

Both simulation and experimental results revealed that the separation performance was the best when *D_x_*/ *D_y_* =  1, and was proportional to *D_p_*/*D_y_*, as described in Figure 3g. The elucidation of this effect is important for the separation of particles at the nanoscale, as observed in Figure 3h. When separating nanoparticles of 50 and 100 nm, they should be first concentrated in a condenser, so as to achieve good separation effect in the following sorter module.

Quantitative evaluation indicates that DLD is suitable for nano‐scale particle separation with a processing throughput of ≈2.0 µL min^−1^.^[^ ^122^ ^]^ Wunsch et al.^[^ ^123^ ^]^ determined the resolution of DLD separation as 20 nm, resulting in a maximum processing throughput of 0.2 nL min^−1^ and a pillar gap (G) of 25 nm. Furthermore, it requires a high‐precision extreme ultraviolet photoetching machine and experts with specialized technology. To address the throughput limitation, Smith et al. proposed the parallelization of 1024 DLD chips to increase the throughput. Exosomes with yields of up to ≈50% were obtained from serum and urine samples. They then performed RNA sequencing on nanoDLD and UC isolated EVs from serum samples of prostate cancer (PCa) patients, and found a higher gene expression correlation in repeats of nanoDLD isolated EVs, which were enriched in miRNA and had reduced rRNA. This approach also successfully detected previously reported RNA markers of aggressive PCa, demonstrating how these nanoparticles carrying biological information, coupled with DLD microfluidic separation technique, can improve diagnosis accuracy and monitoring.^[^ ^124^ ^]^ Shape of the pillar also matters. Hyun et al.^[^ ^125^ ^]^ explored a variety of shapes, discovering that pillar shape can affect *D_c_*. Zhang et al.^[^ ^126^ ^]^ proposed a general equation for defining *D_c_*:

(7) $$D_{c}=\alpha G\varepsilon^{\beta }$$

α and β are the dimensionless geometric coefficients for different pillar shapes. Recently, Razaulla et al.^[^ ^42^ ^]^ reported a new pillar array structure, which consists of 6 hexagonal pillars as a unit, called hexagonal arranged triangle (HAT) geometry. Under the condition of θ < 7°, the structure exhibits a smaller particle separation capability as compared with other published cylindrical parallelogram DLD arrays, and the larger space reduces the risk of clogging and improves processing throughput.

DLD technology is simple to operate, has high precision, and exhibits high‐resolution separation in particle manipulation larger than submicron. However, DLD has its limitations including difficult chip fabrication, easy clogging, and low processing throughput.^[^ ^105^ ^]^

#### 3.2.2 Inertial Microfluidics

In traditional microfluidics, fluid inertia is negligible, and flow predominantly resides in the Stokes flow regime (*Re* ≪ 1). In contrast, inertial microfluidics operates under conditions where flow inertia is significant (1 < *Re* < 100). Especially, when the *Re* is greater than 1, the inertial term becomes more important than viscous. It produces a flow line with a normal (vertical) component to the flow, resulting in flow instability (vortices, oscillations).^[^ ^127^, ^128^ ^]^ Such conditions in inertial microfluidics give rise to a range of nonlinear flow phenomena, notably characterized as inertial migration and secondary flow.

**Figure 4:**
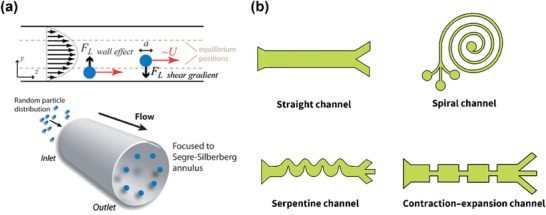
Inertial lift force in straight channel and different channel designs. a) Two forces perpendicular to the direction of flow determine the equilibrium position of the particles. In the case of a cylindrical pipe, randomly distributed particles focus on a ring located between the center of the pipe and the wall. Adapted with permission.^[^ ^131^ ^]^ Copyright 2009, Royal Society of Chemistry. b) Four main inertial microfluidic channels developed. The geometry of the straight channel is simple and easy to manufacture; The Spiral channel can generate continuously varied Dean drag force so that particles of different sizes occupy different focusing positions; The Dean drag force in the serpentine channel varies periodically, facilitating the focusing of particles of different sizes; The contraction‐expansion array structures in the contraction‐expansion channel induces a cross‐sectional Dean flow that differentiates the focusing positions for particle separation. Adapted with permission.^[^ ^132^ ^]^ Copyright 2022, Royal Society of Chemistry.

Inertial migration refers to the behavior observed when dispersed particles at the entrance of a straight channel undergo lateral migration toward several equilibrium positions over a sufficient distance. This migration phenomenon stems from two primary inertial effects: 1) the shear gradient lifting force (*F_LS_*), which is a consequence of the curvature in the fluid velocity distribution interacting with particles, prompting their movement away from the channel centre; 2) Wall lift (*F_LW_*), which originates from the interactions between the flow field of suspended particles and adjacent walls, effectively repelling the particles toward the walls.^[^ ^129^, ^130^ ^]^ In general, the particle is much smaller than the channel size, so the net inertial lift force (*F_L_*) can be described as:

(8) $$F_{L}=\frac{\rho_{f}U^{2}d^{4}}{H^{2}}\;f_{L}\left(Re,x\right)$$

where *U* stands for the average flow velocity, *H* corresponds to the hydraulic diameter, while *f_L_* signifies the non‐dimensional lift coefficient. Importantly, *f_L_* varies with the *Re* and is also influenced by the normalized cross‐sectional position, denoted as (*x*). Secondary flow often occurs in curved or obstructed channels. If the particle is in a curved channel, at any position, the fluid momentum in the centre and near wall regions within the curvature is inconsistent, which results in a radial pressure gradient. Besides, for both sides of the particle, two opposite rotating flows are formed, becoming Dean vortices, which can change the inertial equilibrium position by applying an additional viscous drag force to the particle perpendicular to the main flow stream.^[^ ^128^ ^]^ **Figure** **4**a showed how particles are fixed in specific flow lines.

Current developments in inertial microfluidics have led to the categorization of structures into four main types, as depicted in Figure 4b. The straight channel type primarily utilizes Dean drag force (DDF) and *F_L_* for particle manipulation. In this configuration, the inertial focusing position of particles is influenced by their size. Larger particles tend to focus closer to the centre of channel, while smaller particles gravitate toward the channel walls. This size‐dependent positioning forms the foundational principle for particle focusing and separation.^[^ ^133^, ^134^ ^]^ Hur et al.^[^ ^135^ ^]^ have successfully utilized this structure to focus and purify adrenal cortical progenitor cells from mouse adrenal digestion. However, for nano‐sized particles, the required focusing length localize in a range from 1 m to 1 km, which becomes impractical.^[^ ^45^ ^]^ To this end, Mutlu et al. proposed an oscillatory inertial focusing method, which successfully extended the separation ability of inertial microfluidics to the submicron scale (as small as 500 nm). Unlike conventional steady flow microfluidics, oscillatory microfluidics switches flow direction at high frequency. Due to the symmetry of the velocity field along the flow axis, the direction of the inertial lift acting on the particle is unchanged when the flow direction is switched. By exploiting this symmetry, the length used for focusing can be extended infinitely without increasing the actual length of the channel.^[^ ^136^ ^]^

The spiral channel structure is impacted by a combination of forces: the DDF, *F_L_*, and, in the case of viscoelastic fluids, the elastic force. This combination enhances the structure's capability in particle separation. However, the purity of separated particles using this method has been a concern, so Dean flow fractionation (DFF) was developed to enhance separation accuracy by introducing a sheath flow. This modification in flow dynamics results in smaller, unfocused particles remaining in the original sample flow, while larger particles, influenced more significantly by *F_L_* and DDF, migrate into the sheath flow.^[^ ^137^ ^]^ This approach has recently been adapted to straight channel structures, achieving high separation efficiency with a notable 97.8% purity in platelet isolation from blood. It can also be applied to various particle separation tasks, such as extracellular vesicles or E. coli, and is poised to become a powerful tool for size‐based particle separation in diverse scientific fields, such as physics, biology, and biomedicine.^[^ ^138^ ^]^

In contrast to spiral channels, which have a consistent curvature direction, serpentine channels alternate in curvature direction, reflecting the non‐steady motion of the particles within. Such accumulation of non‐steady states can lead to unpredictable and counterintuitive particle behavior. Di Carlo et al.^[^ ^139^ ^]^ explored the effects of this channel structure on particle dynamics. They found that symmetrical serpentine channels tend to reduce the equilibrium positions of particles, whereas asymmetric serpentine channels further decrease these positions, effectively removing larger particles.

Contraction‐expansion channel represents a more complex design, subjecting particles to a variety of forces: DDF, *F_L_*, planar vortex, and elastic force in the case of viscoelastic fluids.^[^ ^132^ ^]^ Park et al.^[^ ^140^ ^]^ revealed that the ordered distribution of particles is related to their Re. A mismatch in the trajectories between particles and the surrounding fluid elements, especially in regions of transition, leads to a lateral shift in equilibrium positions. This shift is affected by both particle size and flow velocity. With the Re number ranging from 63 to 91, larger polymer particles (≈15 µm) align along the central outlet line, while smaller particles (≈7 µm) travel toward the channel walls.

Inertial microfluidics offers advantages, such as simple operation and high processing capacity, because it does not require built‐in structures within the channel.^[^ ^132^, ^141^, ^142^ ^]^ However, the forces used to manipulate the particles are relatively weak and secondary flows dominate, making it difficult to achieve precise separation accuracy down at the nano scale.

#### 3.2.3 Microfluidic Filtration

Microfluidic filtration (MF) is similar to narrow ultrafiltration (UF) and tangential flow filtration (TFF) in microfluidic chips, with all three methods using porous membranes of varying pore sizes for separation. In the context of nanoscale particles, dead‐end filtration, akin to narrow UF, demands high pump pressure. This high pressure, while effective in some contexts, can potentially damage delicate particles and frequently leads to clogging issues. However, this method is generally considered feasible in terms of achieving a desirable level of purity. On the other hand, TFF, characterized by longer separation paths or cycles, typically necessitates an extended processing time. Despite these challenges, MF has demonstrated successful applications in separating EVs,^[^ ^143^, ^144^ ^]^ biomacromolecule,^[^ ^145^ ^]^ and nanoparticles.^[^ ^108^ ^]^

**Figure 5:**
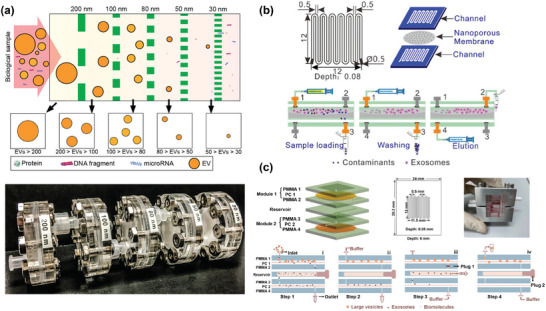
Representative examples of MF techniques. a) Theoretical schematic of the continuous separation of exosomes of different sizes by the ExoTIC device and the design of the overall device. Filters with different pore sizes were realized in series, and the highly modular equipment could meet individual separation requirements. Reproduced with permission.^[^ ^144^ ^]^ Copyright 2017, American Chemical Society. b) A microfluidic TFF device was composed of two PMMA plates with serpentine channels sandwiched with a nanoporous membrane. After loading the sample, those particles with sizes smaller than the membrane pore size were collected first, and then particles larger than the membrane pore size remaining in the microfluidic channel were collected by buffer solution. Reproduced with permission.^[^ ^146^ ^]^ Copyright 2021, Elsevier. c) Microfluidic TFF strategy for parallel modules. The setup of module units and sample handling are similar to the system in (b). This strategy provided finer separation capabilities and higher throughput, with an increased buffer recoil step to obtain the middle fraction. Reproduced with permission.^[^ ^147^ ^]^ Copyright 2023, Elsevier.

Liu et al.^[^ ^144^ ^]^ developed a highly modular MF strategy (ExoTIC) for obtaining different types of EVs (**Figure** **5**a). Filter modules with different pore sizes can be assembled to enrich and separate particles of different sizes for continuous operation. Compared to traditional UC methods, its production capacity has increased by ≈4–1000 times, and the same modules can be paralleled to increase their processing throughput. The platform has been successfully applied to separate and collect EVs of varying particle sizes from plasma, urine, and other fluids. It has been shown that the protein and microRNA levels derived from EVs correlate well with the gold standard ultracentrifugation, demonstrating the device's broad applicability in cancer and other diseases. Additionally, its ability to achieve high‐yield separation from small sample sizes enables sensitive EV‐based proteomic and transcriptomic biomarker detection for downstream point‐of‐care applications.

Figure 5b,c showed the TFF strategy scaled down to a chip. Han et al.^[^ ^146^ ^]^ established serpentine channels on both sides of the nanopore membrane to isolate exosomes from human plasma and derived from HeLa cells (Figure 5b). The cleaning efficiency of protein pollutants was greater than 90%, and the recovery rate was greater than 80%, meanwhile no clogging was observed. However, the separation time of exosomes was ≈3 h for 100 mL sample after 10‐times dilution. To improve efficiency, Hua et al.^[^ ^147^ ^]^ established a double tangential flow filtration strategy (Figure 5c), which realized multistage filtration using series of different nanopore membranes, showing better purity (82.8%) and recovery rate (77.8%) than UC, and a processing time of ≈2 h.

MF can easily control the separation function by adjusting membrane pore size. However, for all filtration methods, the pollution and system pressure caused by membrane clogging can have an impact on the final particulate product. Although TFF is able to mitigate these effects to some extent, its negative effect on the biological integrity of particles cannot be ignored.

#### 3.2.4 Pinched Flow Fractionation

**Figure 6:**
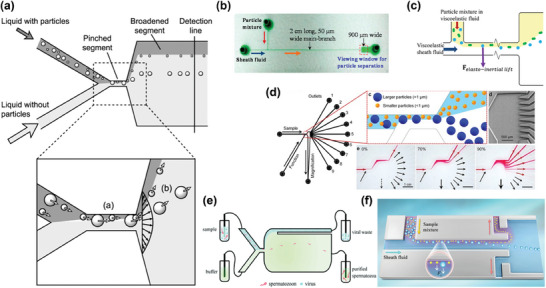
Representative examples of pinched flow techniques. a) PFF continuously introduces particle and non‐particle liquids from each inlet into microchannels that converge in the Pinched segment, where the particles are focused, similar to FlFFF. Subsequently, at the junction of the Pinched segment and Broadened segment, pinched pressure is released, and the force toward the centre of the microchannel is mainly exerted on the larger particles through the diffusion flow profile, while the force toward the sidewall is mainly exerted on the smaller particles, so that the differences between particles are amplified in motion until equilibrium is reached. Reproduced with permission.^[^ ^148^ ^]^ Copyright 2004, American Chemical Society. b) The iPFF strategy lengthens the pinched segment to increase the inertial focus of the particles, so the system could withstand higher processing speeds to achieve the same separation effect. Reproduced with permission.^[^ ^150^ ^]^ Copyright 2015, American Chemical Society. c) eiPFF further introduced elastic forces to iPFF, providing a separation effect between rigid and elastic particles. Reproduced with permission.^[^ ^151^ ^]^ Copyright 2015, American Chemical Society. d) Nine exits and a magnifying channel were designed to more finely separate EVs of different range sizes. Reproduced with permission.^[^ ^152^ ^]^ Copyright 2017, Springer Nature. e) The traditional PFF structure could effectively separate sperm and virus particles with large differences. Reproduced with permission.^[^ ^113^ ^]^ Copyright 2021, Royal Society of Chemistry. f) The structural design of using reverse flow to enhance particle separation in conventional iPFF could separate particles at very low Re and flow rate ratios to significantly improve the processing throughput. Reproduced with permission.^[^ ^153^ ^]^ Copyright 2023, Royal Society of Chemistry.

Pinched Flow Fractionation (PFF) achieves compression by introducing another flow line and setting a narrow channel (Pinched segment) at the intersection of the two flow lines. As shown in the **Figure** **6**a, the force to the center of the microchannel is mainly exerted on larger particles through the diffusion flow profile. While the force toward the side wall is mainly applied to smaller particles, the minor differences of particle position in the clamping section are subsequently amplified significantly in the widened segment, resulting in the perpendicular separation of particles relative to the flow direction according to their size.^[^ ^148^ ^]^ Till now, PFF has been mainly utilized for bioparticle separation.^[^ ^31^, ^143^, ^149^ ^]^

Initial studies on Pinched Flow Fractionation (PFF) demonstrated limited throughput (70–560 µL h^−1^).^[^ ^148^ ^]^ Subsequently, Lu et al. introduced inertia‐enhanced PFF (iPFF, Figure 6b), utilizing flow‐induced inertial lift forces by extending the clamp segment in PFF, thereby enhancing processing throughput.^[^ ^150^ ^]^ Further innovation was to introduce a viscoelastic solution to iPFF, termed elasto‐inertial pinched flow fractionation (eiPFF, Figure 6c). This method improved particle throughput and separation resolution due to the force exerted on particles in the viscoelastic sheath fluid, directing them closer to the side wall.^[^ ^151^ ^]^ Another approach, developed by Shin et al.,^[^ ^152^ ^]^ focused on particle behavior in the pinched segment (Figure 6d), facilitating the separation of extracellular nanovesicles and apoptotic bodies by size. Nevertheless, PFF proved particularly effective for applications that separate particles with significant size differences. For instance, Hamacher et al. successfully employed conventional PFF structures (Figure 6e) to separate viruses from semen, leveraging the considerable size difference and asymmetric shape of sperm for enhanced separation.^[^ ^113^ ^]^ More recently, Wang et al. developed a reverse flow enhanced inertia pinched flow fractionation (RF‐iPFF, Figure 6f),^[^ ^153^ ^]^ which separates particles by size using inertial lift and counter current flows, significantly enhancing particle separation efficiency and achieving ≈10‐fold improvement in throughput.

PFF is simple to design and fabricate, and it has various applications in nanoparticle separation. However, this technique is highly dependent on differences between the separated particles, and its processing throughput is relatively low.

#### 3.2.5 Viscoelastic Microfluidics

**Figure 7:**
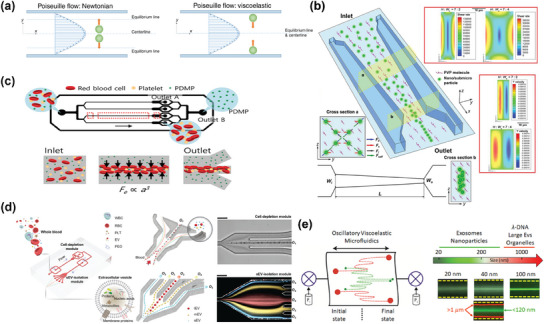
Principle and representative examples of viscoelastic microfluidics. a) Unlike the inertial microfluidics in Newtonian fluids, in viscoelastic fluids, inertial and elastic effects together determine the focusing position of the particles, resulting in a faster rate of migration to the center of particles with larger particle size, thus achieving particle size dependent particle separation. Adapted with permission.^[^ ^116^ ^]^ Copyright 2020, Springer Nature. b) Viscoelastic microfluidics with a gradually shrinking cross‐section microchannel structure. The distribution on the channel cross‐section changes in the microchannel as the cross‐section gradually shrinks, making it easier for the particles to be driven to the center of the previous channel. Reproduced with permission.^[^ ^158^ ^]^ Copyright 2021, John Wiley & Sons. c) Microvesicles in the blood were separated using a parallel strategy, in which large blood cells are focused while microvesicles are separated and collected from both sides. Adapted with permission.^[^ ^159^ ^]^ Copyright 2020, John Wiley & Sons. d) The device comprises two sequential modules: a cell depletion module and a sEV (small, <200 nm) isolation module. Blood components, including white and red blood cells, and PLTS, were first removed from outlet O_1_, followed by a downstream flow of cell‐free blood samples into the sEV separation module, where lEV (large, >800 nm) and mEV (medium, 200 nm) were collected from outlet O_2_ and sEV is collected from outlet O_3_. Reproduced with permission.^[^ ^160^ ^]^ Copyright 2023, American Association for the Advancement of Science. e) Periodic oscillations produce oscillating flows, and the elastic forces exerted on the particles cause them to migrate laterally, ultimately leading to their focusing. Reproduced with permission.^[^ ^117^ ^]^ Copyright 2020, American Chemical Society.

As mentioned in the eiPFF technique,^[^ ^151^ ^]^ the application of viscoelastic forces exploits the elastic properties of non‐Newtonian fluids in microfluidics to manipulate particles. Viscoelastic microfluidics utilizes the elastic forces caused by normal stress imbalance in viscoelastic fluid flow.^[^ ^154^ ^]^ The induced inertial lift acting on the particle can be divided into wall lift (*F_W_*) and shear gradient lift (*F_SG_*) which affects cross‐flow motion of particles:^[^ ^155^ ^]^

(9) $$F_{D}=3\pi \mu_{f}\alpha \left(v_{f}-v_{\rho }\right)$$

(10) $$F_{iL}=F_{W}+F_{SG}=C_{iL}\rho d^{4}{\dot{y}}^{2}$$

(11) $$F_{E}=-2C_{eL}\eta_{p}d^{3}\lambda \nabla {\dot{y}}^{2}$$

where α is particle size in diameter, μ_*f*_ is fluid viscosity, *v_f_* is fluid velocity, and *v*_ρ_ is particle velocity; *C_iL_* is nondimensional inertial lift coefficient, ρ is fluid density, *d* is particle size, and $\dot{y}$ is shear rate; *C_eL_* is nondimensional elastic lift coefficient, η_*p*_ represents the contribution of polymers to the solution viscosity, and λ is the fluid relaxation time.^[^ ^37^ ^]^ In viscoelastic fluids, the inertial effect and the elastic effect together determine the focusing position of particles, resulting in their migration toward the center of the channel with a migration rate proportional to particle size, which generates transverse gaps between different particles. A comparison with inertial fluids makes it easier to understand the different focusing behaviors of the two technologies in microfluidic channel (**Figure** **7**a).^[^ ^116^ ^]^ This technology has been adopted for particle separation and manipulation.^[^ ^37^, ^116^, ^156^, ^157^ ^]^

The focusing effect of viscoelastic microfluidics is affected to some extent by various parameters. Fan et al. applied a microchannel with a progressively shrinking cross section and high aspect ratio to enhance the hydrodynamic force and change the direction of the force (Figure 7b).^[^ ^158^ ^]^ This method can effectively focus particles ranging in size from 500 to 860 nm. Owing to the natural viscoelasticity of plasma, it has been used to isolate EVs and exosomes from blood. Nam et al.^[^ ^159^ ^]^ developed a parallel viscoelastic microfluidic system (Figure 7c), and achieved microvesicle separation with an efficiency ≈4.8 times that of centrifugation. The system also exhibits excellent biocompatibility owing to the absence of external field. To refine this approach, Meng et al. introduced a dual‐module viscoelastic fluid system to process whole blood, remove cells and isolate exosomes (Figure 7d).^[^ ^160^ ^]^ The method achieved an 87% recovery rate and over 97% purity of isolated exosomes, demonstrating advantages such as low cost and high efficiency. More importantly, they confirmed that blood‐derived sEVs purified using this microfluidic method and conventional UC had similar protein compositions, as shown by mass spectrometry and proteomic analysis, indicating that the method effectively preserves the information carried by sEVs. Furthermore, they validated that the concentration and size distribution of sEVs isolated from the blood of 20 healthy donors and 20 cancer patients were consistent with the gold standard UC method. When combined with downstream analyses, such as surface plasmon resonance‐based detection for accurate disease‐specific sEV monitoring, this method becomes a versatile tool for clinical applications.

Similar to the oscillatory inertial focusing method mentioned in 3.2.2, Asghari et al developed an oscillatory viscoelastic microfluidics (Figure 7e)^[^ ^117^ ^]^ to separate p‐bodies from biofluids (in the micrometer range) with a particular focus on lambda‐DNA (in the sub‐micrometer) and extracellular vesicles (in the nanometer range). During the oscillation period, large particles gather near the channel wall, and smaller particles get closer to the central flow line. This phenomenon of particle separation can be explained by the introduction of blockage ratio (*a_p_*/*D_n_*, where *a_p_* is the particle size and *D_n_* is the hydraulic diameter of a microchannel).

Viscoelastic microfluidics has shown promising applications, especially in the field of biomedicine, due to its advantages including label‐free, simple, biocompatible, and capable of particle manipulation at multiple size levels from micron to nanometer.^[^ ^116^, ^159^ ^]^ However, in addition to some viscoelastic biological samples such as blood, the introduction of additional viscoelastic mediators may complicate subsequent processing, potentially contaminating samples from various aspects, especially bioactive particles. Moreover, the processing throughput of viscoelastic media is smaller than that of Newtonian fluid media, otherwise stronger pressure is required, which may also bring potential particle hazards.

### 3.3 Active Microfluidic Techniques Methods

Active microfluidic separation techniques, encompassing acoustic, electric, optical, and magnetic force fields, have been extensively applied in the manipulation and separation of nanoparticles, including EVs and biomacromolecules.^[^ ^31^, ^37^, ^44^, ^45^, ^91^ ^]^ These methods, mediated by external fields in microfluidic environment, facilitate the manipulation of smaller particles and broaden the scope of applications based on additional particle properties like charge, shape and magnetism.^[^ ^90^ ^]^ Notably, they enable surface‐specific labelling strategies that cater to charge and biological particle properties, thus offering enhanced possibilities for differentiating and separating particles of similar sizes.

#### 3.3.1 Acoustofluidics

Acoustofluidics integrates acoustic waves with microfluidics to manipulate particles. The acoustic frequencies and power used are akin to those in ultrasonic imaging, tailored to prevent damage to biological particles.^[^ ^161^ ^]^ Consequently, this technology has been increasingly utilized in the biomedical field for the separation, purification, and detection of various particles, including EVs, viruses, and biomacromolecules.^[^ ^162^, ^163^, ^164^, ^165^, ^166^ ^]^

**Figure 8:**
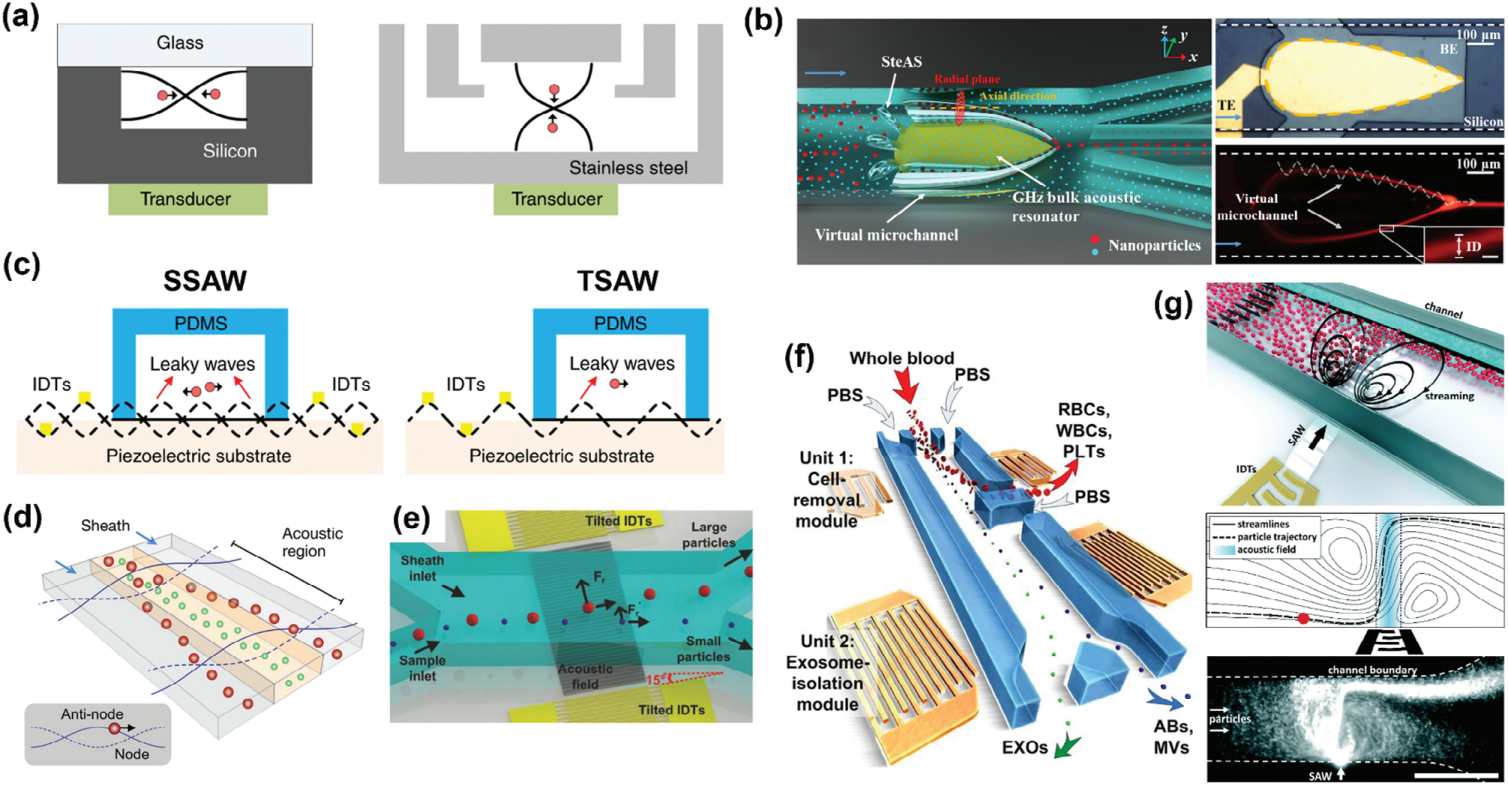
Structure, principle and representative examples of acoustofluidics. a,c) The state of the acoustic wave generated by BAW, SSAW and TSAW in the channel and the force on the particle. Adapted with permission.^[^ ^167^ ^]^ Copyright 2019, Springer Nature. b) The new conical BAW structure avoids the risk of clogging by building virtual channels, while the high‐throughput jet focus allowed the use of larger acoustic wave frequencies with as little damage to particles as possible. Adapted with permission.^[^ ^169^ ^]^ Copyright 2022, American Association for the Advancement of Science. d) In SSAW, the particles are subjected to a radiant force proportional to the particle volume and migrate toward the pressure node, so larger particles move more quickly to the pressure node, transferring to the sheath stream for elution. The higher the flow rate, the longer the processing distance is required, so the processing throughput is limited. Reproduced with permission.^[^ ^173^ ^]^ Copyright 2015, American Chemical Society. e) Another SSAW device structure given an oblique Angle between the SSAW and the microfluidic vector, reducing the number of sheath streams to 1. Reproduced with permission.^[^ ^177^ ^]^ Copyright 2017, John Wiley & Sons. f) Series strategy of SSAW. The cell removal module at the front first separates blood components with diameter bigger than 1 µm, including red blood cells, white blood cells, and platelets, and then exosomes were collected with more than 99% purity by higher frequency acoustic waves and other particles larger than 110 nm. Reproduced with permission.^[^ ^178^ ^]^ Copyright 2017, National Academy of Sciences. g) a TSAW strategy used the vortex formed by the combination of acoustic flow and acoustic radiation force in the microfluidic channel to focus particles on continuous flow. Particles with different particle sizes were subjected to different forces in the direction of the flow line during the escape process, so they focus on different positions. Reproduced with permission.^[^ ^179^ ^]^ Copyright 2017, Royal Society of Chemistry.

Acoustofluidic techniques can be categorized based on acoustic field generation and mediation into Bulk Acoustic Waves (BAWs) and Surface Acoustic Waves (SAWs).^[^ ^167^ ^]^ BAWs (**Figure** **8**a) are generated when the entire piezoelectric material vibrates under the thickness expansion and thickness shear modes. The reflection at the material/fluid interface generates standing waves.^[^ ^168^ ^]^ However, BAWs’ application in manipulating nanoparticles has been limited due to heat generation in high impedance microfluidic materials (like silicon, glass, or stainless steel), which can adversely affect biological particles and challenges in achieving the required wavelengths for nano‐sized particles. A recent innovative design (Figure 8b) has significantly broadened the utility of BAW for nanoparticle manipulation.^[^ ^169^ ^]^ It employs a new vibration mode and equipment structure to create a plane of acoustic vortex orthogonal to lateral flow. This arrangement forms a series of high‐speed rotating micro‐vortices, generates strong resistance and establishes a virtual microchannel without actual microstructures. This design mitigates the risk of biological particle damage due to channel blockages and solid structures such as filters or pillars. Furthermore, in combination with DLD, this technique demonstrated impressive recovery efficiencies of 92.6%, 85.6%, and 54.5% for 300, 200, and 150 nm polystyrene particles, respectively. Additionally, the direct purification of exosomes smaller than 150 nm from plasma indicated its potential in nanoparticle manipulation, particularly for biological particles.

SAW involves vibrations occurring solely on the surface of an elastic material. It is known for its high precision and ease of miniaturization, attributed to the external mediation of sound waves. SAW generation relies on an interdigital transducer (IDT), with the wavelength determined by the IDT finger width and spacing. When a sinusoidal AC signal is applied to the IDT, the inverse piezoelectric effect induces subtle mechanical deformation on the piezoelectric substrate's surface. This deformation induces a mechanical acoustic wave propagating along the direction of the surface deformation.^[^ ^170^, ^171^ ^]^

According to the wave source, SAW can be divided into Standing SAW (SSAW) and Traveling SAW (TSAW) (Figure 8c).^[^ ^165^, ^172^ ^]^ SSAW involves the placement of two IDTs on either side of a microfluidic channel. Lee et al. showed general behavior of particles in SSAW (Figure 8d),^[^ ^173^ ^]^ underscoring the importance of using high ultrasonic frequencies and ensuring efficient energy transfer for manipulating nanoparticles with SSAW. Following optimization, they achieved ≈90% yield for isolating EVs smaller than 200 nm. In their system, interleaving sound waves form nodes, which affect particle behavior to varying degrees under different conditions, and the following formula provides a more specific description:^[^ ^174^ ^]^

(12) $$F_{R}=-\left(\frac{\pi^{2}{P_{O}}^{2}d^{3}\beta_{f}}{12\lambda }\right)\varphi \left(\beta ,\rho \right)\sin\left(2kx\right)$$

(13) $$\varphi \;\left(\beta ,\rho \right)=\frac{5\rho_{P}-2\rho_{f}}{2\rho_{p}+\rho_{f}}\;-\frac{\beta_{P}}{\beta_{f}}$$

where *P_O_* is the acoustic pressure, β is the compressibility, ρ is the density, λ is the wavelength, *k* is wavenumber of the acoustic waves, *x* is the distance from a pressure node, and the subscripts *f* and *P* represent fluid and particle, respectively. Acoustic radiation force (*F_R_*) is a directional force acting on a particle, which is determined by the acoustic contrast factor. The directional forces are determined by the difference in density and compressibility between the particle and the fluid. When the contrast factor is positive (φ > 0), particles migrate toward the pressure node; when φ < 0, particles move toward the antinode.^[^ ^173^ ^]^ According to Equation (12), the acoustic manipulation of particles is not only limited to particle size, but also affected by their properties (density and compressibility), indicating the potential to manipulate particles of different sizes and different physical or mechanical properties.^[^ ^175^, ^176^ ^]^ Wu et al. introduced a tilt Angle into SSAW microfluidics (Figure 8e), to successfully separate particles with diameters of 900, 600, 220, and 110 nm with over 90% purity.^[^ ^177^ ^]^ Subsequently, they connected modules to devise an integrated method for automatically separating exosomes or other EVs from undiluted blood samples (Figure 8f). The initial module in this setup was employed to eliminate larger blood components, followed by a dedicated exosome separation module for isolating extracellular vesicle subsets. This technique not only differentiated EVs and exosomes but also aimed to separate non‐exosome particles and soluble proteins from exosomes, which share similar sizes but exhibit different acoustic shrinkage factors.^[^ ^178^ ^]^

TSAW are generated using IDTs positioned on only one side of the microfluidic channel. It is predominantly employed in microfluidics for enrichment, concentration, or trapping of nanoparticles, which is ascribed to vortex acoustic streaming within the channel. As demonstrated by Collins et al.^[^ ^179^ ^]^ in Figure 8g, the trajectory of a particle in TSAW is determined by the interaction between the drag caused by the acoustic flow and the primary acoustic radiant force (PRF) or secondary acoustic radiant force (SRF), which can be expressed by the dimensionless coefficient dominant force (*K_tr_*) introduced by Skowronek et al.:^[^ ^180^ ^]^

(14) $$K_{tr}=\frac{2\pi R_{p}}{\lambda }\;$$

where *R_p_* is particle radius and λ is sound wavelength. When *K_tr_* < 1, the drag force caused by acoustic flow is dominant, particles are trapped in the vortex. Conversely, when *K_tr_* > 1, particles are driven by PRF or SRF and away from IDT.^[^ ^172^ ^]^ Collins et al. constructed an “acoustic trap” using strong acoustic currents near highly focused high‐frequency TSAW to robustly manipulate nanoscale particles.^[^ ^179^ ^]^ This work demonstrates the ability to continuously differentiate 100, 300, and 500 nm particles with increased acoustofluidic throughput. Nevertheless, its applicability to biological particles is unknown.

Acoustofluidics in particle manipulation primarily includes BAW, SSAW, and TSAW. Each wave type is characterized by its wave position and generation mode, influencing distinct fluid behaviors. Commonly, these technologies offer benefits such as non‐contact operation, biocompatibility, high controllability, and versatility. However, limitations include lower throughput, the requirement for shorter wavelengths to manipulate nanoparticles effectively, and potential heat generation that may affect the stability of biological and soft particles.^[^ ^167^ ^]^ Furthermore, it is worth to recognize that acoustic manipulation relies on not just particle size but also on other acoustic properties of the particles, which opens avenues for its further development and applications.^[^ ^161^ ^]^

#### 3.3.2 Microfluidic Chip Electrophoresis

Microfluidic Chip Electrophoresis (MCE) represents an advancement in electrophoresis technology, integrating microfluidics with methods such as Gel Electrophoresis (GE), Capillary Electrophoresis (CE), Dielectrophoresis (DEP), and Electric Field Flow Fractionation (ElFFF).^[^ ^181^, ^182^, ^183^ ^]^ Since most biochemical particles have different charges or purifiable properties, they have various applications in biological macromolecules/particles, and AuNP.^[^ ^184^, ^185^, ^186^, ^187^ ^]^ In general, the behavior of charged particles in an electric field is easy to predict, due to electrostatic forces, charged particles are pulled toward electrodes with opposite charges. Particles with more charges are more severely affected, whereas uncharged particles are not affected.^[^ ^188^ ^]^

In CE, if the solution around the particle is considered to exert frictional resistance as it moves, the mobility at net equilibrium is expressed for spherical particle as:^[^ ^189^ ^]^

**Figure 9:**
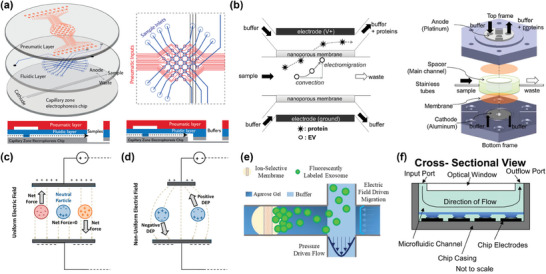
Representative examples of microfluidic chip electrophoresis‐based separation. a) Fully integrated multi‐layer microfluidic CE analyzer for amino acid separation analysis by electrophoresis and fluorescent labeling. Reproduced with permission.^[^ ^192^ ^]^ Copyright 2013, American Chemical Society. b) Microfluidic electrophoresis for separation of EVs and proteins. Both sides of the microfluidic channel were provided with nanopore membranes for screening base on particle size, and the electrophoresis generates a vertical driving force according to their charge. Reproduced with permission.^[^ ^196^ ^]^ Copyright 2016, Elsevier. c) and d) are the forces of particles in a uniform electric field and a non‐uniform electric field, respectively. The former is electrophoresis, which can only act on charged particles, and the latter is dielectrophoresis, which can act on dielectric particles no matter it charged or not. Reproduced with permission.^[^ ^44^ ^]^ Copyright 2021, American Chemical Society. e) Electrophoresis driven the exosomes to move into the vertical gel, and other particles were eluted. The correct exosomes were then purified by a gel to remove the cell debris and enriched at ion‐selective membrane. Reproduced with permission.^[^ ^197^ ^]^ Copyright 2017, John Wiley & Sons. f) Multiple multi‐layer DEP microelectrode arrays were installed at the bottom of the microfluidic channel, and alternating electric fields were applied to generate DEP forces on the particles, but not on free plasma protein, so that the particles were trapped to the high field area at the edge of the electrode. After the channel was washed clean, the reverse DEP force was released, and the target particles were collected. Reproduced with permission.^[^ ^200^ ^]^ Copyright 2015, John Wiley & Sons.

(15) μ=6πηRq

where μ is electrophoretic mobility, *q* is net charge, *R* is particle radius, and η is viscosity. CE is highly efficient for small molecule analysis, yet extending its application to nanoparticles presents challenges. This complicates the electrophoresis process, as particle size cannot be considered negligible as with small molecules, which challenges the basic understanding of the electrophoresis process. AuNPs, as hard particles, are more amenable to electric field analysis. Franze et al. enhanced the separation accuracy of AuNPs down to 10 nm using CE.^[^ ^190^ ^]^ Additionally, details regarding the hydrodynamic radius and the mass fraction of individual elements (using ICP‐Q‐MS) or multiple elements (using ICP‐SF/MC‐MS or ICP‐ToF‐MS) can be obtained on a particle‐by‐particle level. Furthermore, SP‐ICP‐MS results serve as a diagnostic tool for refining and validating coupled separation techniques. Bouri et al. achieved the isolation of eight distinct small AuNPs with a 2 nm resolution in size, averaging 5.3, 5.6, and 5.1 nm, through Capillary Zone Electrophoresis (CZE), which demonstrates a high‐precision separation capability.^[^ ^191^ ^]^ On a smaller scale, Kim et al. developed an integrated multilayer microfluidic analyzer for automated sampling and analysis using CE (**Figure** **9**a).^[^ ^192^ ^]^ This system employs lift‐gate microfluidic valve technology and soft lithography to create a microfluidic automaton with a 2D microvalve honeycomb array for efficient biomarker and biological macromolecule analysis. However, the potential heat generation in the electric field and its consequent damage to soft biological particles limit the further applications of CE and Gel Electrophoresis (GE) in nano‐scale particle manipulation. GE's additional shear forces also pose risks to particles.^[^ ^193^ ^]^

Electrophoresis is an effective technique for separating EVs and biomacromolecules, leveraging their physical properties like size and ζ potential.^[^ ^44^, ^194^, ^195^ ^]^ Cho et al. established an electrophoretic migration‐based EVs separation system (Figure 9b),^[^ ^196^ ^]^ achieving an 8‐fold more efficient separation as compared to standard UF, meanwhile maintaining high size selectivity and processing throughput, capable of handling milliliters of samples within an hour. Compared to the immunoaffinity separation method, it better reduces the irreversible destruction of EVs. Western blot and RT‐PCR analyses confirmed that the EV content (surface proteins and RNA) remained intact, making it well‐suited for preparing recovered EVs for further experiments. Testing on plasma samples also demonstrated high recovery and purity in body fluid separation, with nano‐scale impurity levels reduced to half that of commercial precipitation methods. Marczak et al. utilized the ion consumption properties of ion‐selective membranes to create a local transverse field that guides EVs into an agar‐gel (pore size 200–300 nm) and toward a negatively charged cation exchange membrane (Figure 9e).^[^ ^197^ ^]^ This process resulted in concentrated EVs on the membrane surface, enabling the successful enrichment of exosomes with particle sizes ranging from 60–130 nm, while enhancing process throughput (over 70% of incoming exosomes at 150 µL h^−1^ for at least 20 min). Additionally, this microfluidic separation design proved successful with clinical samples, enabling both the isolation and concentration of exosomes, which could facilitate the sensitive detection of key biomarkers for early cancer detection.

DEP differs from the uniform electric field approach used in traditional electrophoresis (Figure 9c). As depicted in Figure 9d, DEP involves the migration of polarizable (dielectric) particles in a non‐uniform electric field. In DEP, both charged and neutral particles become polarized in the non‐uniform electric field and are moved by an unbalanced electrostatic force, allowing for the manipulation of both types of particles.^[^ ^44^ ^]^ The force exerted on a particle in DEP is contingent on the dielectric properties of both the particle and the suspension medium. For uniform spherical particles, the DEP force (*F_DEP_*) can be described as:^[^ ^198^, ^199^ ^]^

(16) $$F_{DEP}=2\pi a^{3}\varepsilon_{m}Re\left[K\left(\omega \right)\right]\nabla |E_{rms}{|}^{2}$$

(17) $$K\;\left(\omega \right)=\frac{\varepsilon_{p}^{*}-\varepsilon_{m}^{*}}{\varepsilon_{p}^{*}+2\varepsilon_{m}^{*}}\;$$

(18) $$\varepsilon_{p}^{*}=\varepsilon_{p}\;-\frac{i\sigma_{p}}{\omega }$$

(19) $$\varepsilon_{m}^{*}=\varepsilon_{m}\;-\frac{i\sigma_{m}}{\omega }$$

where *Re*[*K*(ω)] is the real part of the Clausius–Mossotti (CM) factor, ω is the electrical frequency, and *E_rms_* represents the root mean square of an electric field, ε* is complex permittivity, ε_*p*_ is permittivity of the particle, and σ is conductivity. The CM factor's sign (positive or negative) in DEP determines the movement direction of particles by reflecting the relative magnitude of a particle's permittivity or conductivity compared to the medium. This principle is extensively used for separating nanoparticles and biomolecules, such as viruses and exosomes.^[^ ^44^ ^]^ Ibsen et al. employed DEP to extract nanoparticles from complex biological samples effectively.^[^ ^200^ ^]^ They constructed a multilayer DEP microelectrode array beneath a microfluidic channel (Figure 9f). This array consists of over 1000 electrodes with specific shapes to create a field gradient that can concentrate the highest field region into a ring at each electrode's edge. When the electric field oscillates at an appropriate frequency, the dielectric force draws particles toward the electrode edges. This setup proved effective in processing various particles, including empty liposomes, liposomes filled with cross‐linked human serum albumin, hollow silica shell nanoparticles, and solid nanoparticles composed of prodrug monomers. This is essential to understand the surface properties of those nanoparticles exposed to complex biological fluids, which can affect the in vivo properties of these particles, greatly limiting their therapeutic efficacy. Next, they proceeded to test the ability of the AC electrokinetic (ACE) microarray chip to quickly separate and recover glioblastoma exosomes from undiluted plasma samples. Exosomes were isolated in less than 30 min, and a combination of immunofluorescence assay and RT‐PCR assay confirmed the presence of exosome‐specific external CD63 protein, internal TSG101 protein, and RNA release from the vesicles. This demonstrates the potential for minimally invasive cancer diagnosis, particularly for the rapid isolation of exosomes, associated RNA and proteins, and cf‐DNA biomarkers. This method paves the way for rapid, seamless sample‐to‐result liquid biopsies, treatment monitoring for cancer patients, and ultimately, early disease detection.^[^ ^201^ ^]^ But the reality is, although DEP can interact with neutral particles, particles require prolonged exposure to a non‐uniform electric field to achieve high accuracy, which limits its throughput.

Electricity‐based separation methods have shown exceptional efficiency and resolution in processing hard nanoparticles like AuNP, and hold significant promise for manipulating biological particles with diverse polarization and charge properties. However, there is a continuing concern regarding the potential damage to biological particles caused by direct exposure to electric fields. Furthermore, the direct contact between the sample solution and electrodes, along with the heat generated by the high operating voltages required for some devices, poses additional risks to the integrity of the separation targets.^[^ ^44^ ^]^

#### 3.3.3 Field Flow Fractionation

**Figure 10:**
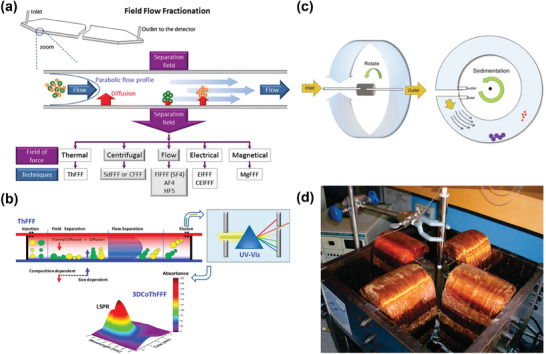
The basic structure, principle and representative examples of field flow fractionation. a) In the general FFF, according to the required particle properties (such as particle size, charge, magnetic, etc.), the corresponding force field is given in the orthogonal direction of the main path, so as to separate particles with different corresponding properties. Reproduced with permission.^[^ ^202^ ^]^ Copyright 2017, Springer Nature. b) A 3D correlated ThFFF (3DCoThFFF) strategy for advanced analysis of nanostructures of novel metal polymers. Orthogonal thermodynamic gradients were constructed on both sides of the microfluidic channels to separate particles. Reproduced with permission.^[^ ^211^ ^]^ Copyright 2023, American Chemical Society. c) Within the SdFFF pathway, particles with different density are allocated among various axial flow directions based on the equilibrium of the exerted centrifugal field and particle dispersion. The centrifugal action leads to the settling of the particles, driven by the multiplication of their effective capacity and the density variance between the particles in suspension and the surrounding fluid. Reproduced with permission.^[^ ^219^ ^]^ Copyright 2020, Elsevier. d) A quadrupole MgFFF technique, similar to the QTOF‐MS technique, was developed to separate and characterize magnetic nanoparticles, which was unique in its ability to determine the magnetic distribution of nanoparticle samples in suspension. Reproduced with permission.^[^ ^218^ ^]^ Copyright 2009, American Chemical Society.

As mentioned in MCE, electric fields can mediate electric field flow fractionation. In fact, Field Flow Fractionation (FFF), a flow chromatography‐based separation method, is operated by integrating different fields with fluid vector within a microfluidic channel to achieve particle separation. Retention and separation in a typical channel result from an externally applied force field oriented perpendicularly to the flow axis, as illustrated in **Figure** **10**a.^[^ ^202^, ^203^ ^]^ The separation mechanism is straightforward. Particles with different molar masses, sizes, or other physical characteristics behave differently under the applied field, thus moving into various speed zones within the parabolic flow profile created by the laminar flow of the mobile phase. Consequently, particles with different characteristics exhibit varied outflow times, similar to “retention times (*t_R_*)”.^[^ ^204^ ^]^ Thus, if the field can exert a force on the target particles, similar structures can be engineered to manipulate particles on the basis of their characteristics. This technique has found applications in separating both biological and non‐biological nanoparticles.^[^ ^204^, ^205^, ^206^, ^207^ ^]^

Regardless of the type of additional force field applied, particles can be uniformly analyzed with a focus on their nanometer size. When considering the vertical application structure for such small particles, it's critical to consider the diffusion effect. As discussed above, the accumulation of particles at the wall of the microfluidic channel creates a significant concentration gradient. This gradient impacts the *t_R_* of the particles, which can be mathematically expressed as:^[^ ^208^ ^]^

(20) $$t_{R}=\frac{t_{0}}{6\frac{D_{f}}{wU_{x}}\left[\frac{1+e^{\frac{-wU_{x}}{D_{f}}}}{1-e^{\frac{-wU_{x}}{D_{f}}}}-\frac{2D_{f}}{wU_{x}}\right]}\;$$

where *t*_0_ is the void time, *w* the height of the channel, *D_f_* is the diffusion coefficient of the particle (as described in Equation (3)), *U_x_* represents the speed of the particle in the height direction of the channel. In addition, similar to the behavior of molecular fragments in a quadrupole, the vector of the applied force field can be periodic. In this scenario, there are three modes where the distance travelled *l* is greater than, equal to, or less than *w*. Each case can be analyzed separately as:

1. When *l* > or < *w*, the particle has time to slide along each wall before the direction of the force reverses or the force applied is not sufficient to move the particle directly through the channel, respectively:
(21) $$t_{R}=\frac{2t_{0}wf}{3|U_{x}|-\frac{|U_{x}{|}^{2}}{wf}}\;$$
2. Where *f* represents the frequency of the force, and when the force is just enough to push the particles through the channel (*l* = *w*):
(22) $$t_{R}=\frac{t_{0}\left|U_{x}\right|}{2wf}\;$$

The types of force include thermal, centrifugal force, flow field force, electric field force, and magnetic field force (Figure 10a). Detailed analyses of these forces were discussed in other comprehensive reviews.^[^ ^205^, ^209^ ^]^

Thermal FFF (ThFFF) utilizes a thermal gradient across microfluidic channels to induce thermophoretic mobility, depending on particle characteristics such as charge, tactile sensation, branching, or size.^[^ ^210^ ^]^ Using this principle, Muza et al. recently developed an innovative 3D correlated ThFFF technique (Figure 10b) that allows high‐resolution tracking of multiple distributions in imidazolium‐containing ionic polymers (IIP)s functionalized AuNPs^[^ ^211^ ^]^ (IIP‐AuNPs). Production and validation of the successfully functionalized IIP‐AuNPs with higher resolution and sensitivity were achieved by combining ThFFF separation combined with local surface plasmon resonance (LSPR) and 2D UV‐VIS spectroscopy detection.^[^ ^211^ ^]^ Smith et al. expanded the scope of ThFFF by combining it with light scattering detectors for various inorganic nanoparticles,^[^ ^212^ ^]^ demonstrating a potent capacity for characterizing and separating particles based on their component forces, chemical composition, and structure (including shape). They also highlighted the influence of the thermodynamic properties of solvents on the shrinkage and branching heat diffusion of the polymer cable. These metal nanoparticles have been widely used in drug delivery, biosensors, and wearable electronics, and their precise detection, separation, and production can significantly enhance the effectiveness of these applications.^[^ ^213^ ^]^

Sedimentation FFF (SdFFF) utilizes gravitational or centrifugal forces for particle separation, with centrifugal force typically generated by channel rotation,^[^ ^214^ ^]^ as the general structure was depicted in Figure 10c. Schallinger et al. demonstrated the effectiveness of SdFFF in biomolecule analysis, achieving the purification of samples containing particles or soluble macromolecules.^[^ ^207^ ^]^ Notably, the technique showed nearly 100% nucleic acid recovery without altering molecular weight or conformation, and displayed excellent biocompatibility. However, limitations in processing throughput restrict the broader application of SdFFF, as illustrated by Saeseaw et al., where the particle size in aggregates increased from 0.19 µm (at 15 min) to 0.38 µm (over 2880 min).^[^ ^215^ ^]^

Flow FFF (Flow FFF) encompasses both symmetric (FlFFF) and asymmetric (AF4) types, employing a crossflow of a second fluid from the eluent, perpendicular to the microfluidic channel. Given that its force source is fluid flow, Flow FFF demonstrates excellent biocompatibility and is versatile enough for almost all particle manipulations.^[^ ^209^, ^216^ ^]^ However, issues related to membrane often pose challenges to particle separation. Electrical FFF (ElFFF), as discussed in the context of MCE, utilizes vertical forces from electrophoresis or DEP. Its advantages include comprehensive sorting based on particle charge, polarity, and mobility. Nonetheless, potential biohazards remain a significant constraint. Magnetic FFF (MgFFF) is particularly effective for magnetic nanoparticles.^[^ ^217^ ^]^ The development of the Quadrupole MgFFF system (QMgFFF) marked a significant advancement. QMgFFF (Figure 10d) employed a quadrupole magnetic field with axisymmetric characteristics to separate various magnetic nanoparticles.^[^ ^218^ ^]^ However, the requirement for particle magnetism restricts its applicability.

The structural versatility of FFF to mediate and combine different types of fields attracts interest in research and industry. However, it usually takes longer to set up the FFF system and process particles.^[^ ^209^ ^]^ In the future, with advanced fabrication method and improved efficiency, multi‐field series can be considered to achieve more powerful particle manipulation.

#### 3.3.4 Magnetophoresis

**Figure 11:**
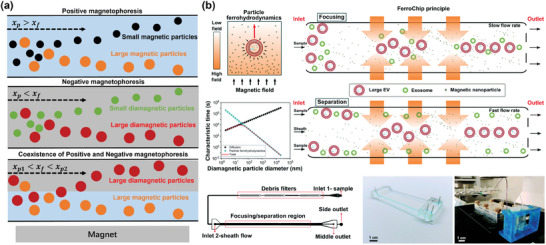
The mode of action of magnetic fluids and the possibility of manipulation for unlabeled diamagnetic nanoparticles. a) Three modes of action of magnetic/diamagnetic particles of different sizes in magnetic/diamagnetic fluids. b) In stable magnetic nanoparticle (≈10 nm) suspension (i.e., ferrofluid), the magnetic susceptibility of unlabeled EVs and exosomes was much lower than that of ferrofluid (***x***_***p***_ < ***x***_***f***_), the pressure difference on the vesicle surface induced by magnetic nanoparticles produces a force proportional to the volume of EV; In the focusing mode, the samples entered the linear microfluidic channel at a slow flow rate, and the symmetrical magnetic field with the minimum central value focused EVs and exosomes toward the center of the microchannel. Following the separation module, the center's faster sheath flow size‐dependent focused the EVs to the center to achieve separation. Reproduced with permission.^[^ ^228^ ^]^ Copyright 2020, Royal Society of Chemistry.

In magnetophoresis, the essential requirement is the presence of an entity responsive to magnetic fields, which could be either particles or the fluid itself. Based on their magnetic field response, materials can be categorized into three types: diamagnetism, paramagnetism and ferromagnetism. Diamagnetic materials, including water and most biological particles, are essentially non‐magnetic. When exposed to a magnetic field, they exhibit only a slight imbalance in orbital electrons, leading to the creation of small magnetic dipoles that oppose the applied field, thereby demonstrating diamagnetism; Paramagnetic materials, such as Chromium (Cr), Manganese (Mn), Oxygen (O_2_), Nitrogen Dioxide (NO_2_), and various oxides, show a weak attraction to magnetic fields. This attraction occurs as the magnetic dipole moments of their atoms or molecules align with the direction of the magnetic field, resulting in magnetic attraction. Ferromagnetic materials, including common iron (Fe), Cobalt (Co), and Nickel (Ni), are unique in their ability to retain magnetization even without a magnetic field.^[^ ^31^, ^220^, ^221^ ^]^ The force acting on these particles in a magnetic field can be mathematically described as:^[^ ^222^ ^]^

(23) $$F_{m}=\frac{\Delta xv}{\mu_{0}}\;\left(B.\nabla \right)B$$

where Δ*x* is the difference between dimensionless volume magnetic susceptibilities of particles and the fluid *x_p_* − *x_f_*, *v* is the volume of the particle, *B* is the applied magnetic field, and μ_0_ =  4π × 10^−7^ *Tesla*.*meter*/*Ampere* is the permeability of free space. A disparity in susceptibility is essential to generate a magnetic force on a given particle. Depending on the susceptibility magnitudes, magnetophoretic force might manifest as either positive or negative. A scenario where Δ*x* ​> 0 will result in positive magnetophoresis, leading the microparticle or cell toward the peak regions of the non‐homogeneous magnetic field. An illustrative case is magnetic microparticles submerged in water. Conversely, when diamagnetic particles (with Δ*x* < 0) are placed in a magnetic environment (like paramagnetic fluids or ferrofluids), the microparticles move toward the lowest intensity areas of the non‐homogeneous magnetic field. This phenomenon is specifically termed negative magnetophoresis. One can observe this phenomenon with polystyrene microparticles in ferrofluids or paramagnetic liquids. These particles are attracted to the weakest gradient zones of the irregular magnetic field, exhibiting the characteristics of negative magnetophoresis. The third situation is *x*_*p*1_ < *x_f_* < *x*_*p*2_, which allows particles of the same size but different magnetism to be separated.^[^ ^223^, ^224^ ^]^ The three particle manipulation modes are shown in **Figure** **11**a, and as described in FFF section, the general structure of magnetophoresis is to establish magnetic fields in the orthogonal directions of microfluidics, with no clear boundary between them. However, these structures present challenges in processing throughput and accuracy.

Although magnetophoresis has proven effective for particles larger than 1 µm, manipulating diamagnetic nanoparticles without labels remains challenging.^[^ ^225^, ^226^, ^227^ ^]^ Magnetic fluids offer a potential solution. Liu et al. developed a “particle ferrohydrodynamics” system,^[^ ^228^ ^]^ integrated with a microfluidic device called the FerroChip (Figure 11b). This setup enabled the continuous, size‐dependent separation of exosomes and EVs from cell culture medium and human serum, achieving recovery and purity rates of 94.3% and 87.9%, respectively, with a dimensional resolution of ≈100 nm.

Since magnetic field does not transfer energy like acoustic field or directly acts on particles through the fluid medium like electric field, the technology has the advantages, such as non‐contact high throughput, low cost, and minimal heat production.^[^ ^220^, ^229^ ^]^ However, without immunoaffinity, its application in biological particles is extremely limited.

#### 3.3.5 Optofluidics

**Figure 12:**
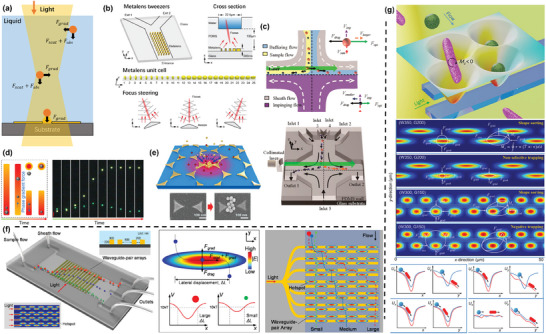
Manipulation of particles by optical filed for separation and its representative examples. a) The force generated by the optical field on particles at different positions in the liquid, and the manipulation of particles using plasma nanostructures. b) The tunable metalens tweezers constituted an array of silicon nanocolumns on the top of the glass substrate. The cross‐section was elliptical, and the shape and size vary with the x position of the nanocolumn. Coherent control technology was used to control the focus for flexible and adjustable optical force generation. Adapted with permission.^[^ ^234^ ^]^ Copyright 2020, Optica Publishing Group. c) Two core inlets and two sheath inlets made up the mainstream, and multi‐layer flows formed a complex crossroads system where smaller particles remain in sample flow 3 and enter exit 1 on the left, while larger particles were pushed into buffer flow 4 and eventually transported to exit 2 on the right. Adapted with permission.^[^ ^237^ ^]^ Copyright 2016, American Chemical Society. d) Transporting individual silver nanoparticles dynamically in two optical line traps using phase‐gradient forces. Reproduced with permission.^[^ ^238^ ^]^ Copyright 2016, American Chemical Society. e) Plasma capture technology enhanced the ability to capture particles. Reproduced with permission.^[^ ^239^ ^]^ Copyright 2016, American Chemical Society. f) The SWANS system evenly distributed the input laser power to the array of waveguide pairs through the beam splitter, set an appropriate distance to facilitate the coupling of light back‐and‐forth between the two waveguides, and ensured sufficient separation between adjacent waveguide pairs to prevent mutual coupling, so as to form the optical lattice. Particles flowing through the lattice were subjected to ***F***_***scat***_ + ***F***_***grad***_ perpendicular to the fluid that produces deterministic lateral displacement. Adapted with permission.^[^ ^240^ ^]^ Copyright 2021, Elsevier. g) ONSA for the manipulation of spherical (*S. aureus*) and rod‐shaped (*E. coli*) bacteria. The optical potential trap was used to separate particles of different shapes according to the particle torque, in which the array parameters are important parameters for the shape sorting of normal biological particles, the non‐selective capture of small biological particles, the shape sorting of normal particles, and the capture of large biological particles. The optical tunability of these parameters did not rely on repeated additive manufacturing, demonstrating the high adaptability of this strategy. Adapted with permission.^[^ ^241^ ^]^ Copyright 2019, American Chemical Society.

Optical manipulation of particles involves controlling particle movement using optically induced electromagnetic fields.^[^ ^230^ ^]^ The interaction of particles with optical waves largely depends on their size. Particles less than the wavelength of optical waves are attracted toward regions of high electromagnetic field intensity as they generate an electric dipole moment responding to the electric field of light.^[^ ^231^ ^]^ The forces involved in optical manipulation typically include the optical gradient force (*F_grad_*), scattering force (*F_scat_*) and absorption force (*F_abs_*), which are described in Equations (24)–(27).^[^ ^232^ ^]^ Actions of these forces and their effects under various conditions are illustrated in **Figure** **12**a.

(24) $$F_{grad}=\frac{2\pi \nabla I_{o}\alpha }{c_{o}}\;$$

(25) $$F_{scat}=\frac{8\pi I_{o}\alpha^{2}\varepsilon_{m}}{3c_{o}\lambda^{4}}\;$$

(26) Fabs=2πIoεmcoλImα

(27) $$\alpha =3V_{p}\left(\frac{\varepsilon_{p}-\varepsilon_{m}}{\varepsilon_{p}+2\varepsilon_{m}}\right)$$

Where ∇*I_o_* is the gradient of light intensity, *c_o_* is the speed of light, λ is the optical wavelength, ε_*p*_ and ε_*m*_ are the permittivity of the particle and suspending medium, respectively, *V_p_* is the particle volume. The positive or negative of α depends on the relative refractive index between the particle and the medium. When α > 0, particles are drawn to the areas of highest intensity, otherwise, particles will move away.^[^ ^233^ ^]^

Optical manipulation introduces light as an external field, which can be precisely controlled using lenses. Yin et al. constructed a structure composed of tunable metalens pillars (Figure 12b),^[^ ^234^ ^]^ where particle manipulation was achieved through coherent control of two backpropagating beams, with controlled relative phase and intensity as light, to trigger different electromagnetic responses from the meta surface. Upon optimization, the system enabled the manipulation of polystyrene particles down to 100 nm as well as metal nanoparticles, particularly AuNPs. AuNPs are known for their unique optical and chemical characteristics, which facilitate plasmonic resonances on local surfaces, making them suitable for diagnostics and biochemical sensing.^[^ ^235^, ^236^ ^]^ Due to their strong light scattering properties, AuNPs are readily manipulated by optical forces in fluids, as describe in Equation (25). Therefore, Wu et al. designed a system for separating AuNP, termed “crossroads” (Figure 12c).^[^ ^237^ ^]^ In this system, AuNPs are confined in a narrow central flow through hydrodynamic focusing on the left side. They slow down upon entering the impact zone, which is crucial for achieving high throughput. After passing through the separation functional region, they are accelerated out of the separation zone. Finally, they are sorted by fluid separation in different directions. This system achieved sorting fidelities of ≥ 92% for 50/100 nm particles, with a resolution of 20 nm, albeit at the cost of processing throughput. Nan et al. achieved an even smaller resolution of 10 nm.^[^ ^238^ ^]^ They employed a spatial light modulator (SLM) to generate an optical trap with adjustable phase‐gradient forces, successfully separating gold and silver nanoparticles (Figure 12d). Moreover, the specific structure can enhance the particle manipulation capabilities of the optical field. Hong et al. constructed a structure based on classical electromagnetic theory with a sharp tip structure to enhance the directly related electromagnetic field (as shown in Figure 12e), capable of capturing and detecting 50 nm AuNP.^[^ ^239^ ^]^

Inspired by DLD and optical focusing actions, Zhao et al. constructed a novel optical nanoparticle separation system called SWANS (Silicon Waveguide‐pair Array‐based Nanophotonic Sorting).^[^ ^240^ ^]^ As shown in Figure 12f, a near‐field optical lattice was created by coupling the waveguide to the back‐and‐forth light in the array. Particles of different sizes undergo repeated trapping and deflection activities through the lattice, and eventually form different trajectories. The system boasts a theoretical resolution of less than 5 nm. It effectively separated particles measuring 200, 300, and 500 nm with a separation efficiency exceeding 95%. Additionally, it proficiently isolated 95 nm Staphylococcus aureus from a mixture of bacteria and nanoparticles, achieving an isolation efficiency of 85.7%. In addition, a similar nanophotonic array strategy also exhibits unmatched sorting performance, that is, sorting particles by shape. Shi et al. reported an optofluidic nanophotonic sawtooth array (ONSA) (Figure 12g).^[^ ^241^ ^]^ The serrated optical field generated by optical coupling enables particles to interact with coupled hot spots, applying different optical torques depending on their shape. When *S. aureus* and *E. coli* flow through ONSA structural points, their motion involves bacterial locomotion, random Brownian motion, and optical forces from adjacent potential wells, leading to rotational movement. E. coli with large aspect ratios escape, while round *S. aure*us is captured, resulting in removal of more than 97% of *E. coli*.

Although the manipulation of metal particles by optofluidic achieves high resolution and precision, the manipulation of other types of particles, especially biological particles, is less effective. While some studies have explored this application, the potential heat production poses biological risks, which is an unavoidable limitation.

### 3.4 Hybrid Microfluidic Techniques

**Table** **1** provides a detailed summary of both passive and active microfluidic techniques, serving as a foundation for their hybridization. Hybrid microfluidic techniques have significantly advanced particle separation at nanoscales, with emerging studies exploring their effectiveness in nanoparticle separation.^[^ ^112^ ^]^ The discussion above centered on techniques applied to various nanoparticles, especially focusing on the forces exerted on particles in fluid, which are crucial for their distinct behavior. While some technologies claim to achieve high processing throughput and separate smaller nanoparticles or achieve higher purity simultaneously, they often employ series or parallel configurations. For instance, inertial microfluidics or viscoelastic microfluidics are frequently combined with pinched flow fractionation.^[^ ^150^, ^160^ ^]^ This section will delve into the hybridization strategies between different techniques, with a particular emphasis on integrating different techniques for particle manipulation. Parallel strategies are generally employed to increase processing throughput using similar techniques,^[^ ^100^, ^242^ ^]^ whereas serial applications of the same technology tend to sequentially alter the forces acting on different particles, aligning with a continuous control strategy.^[^ ^144^, ^178^, ^243^ ^]^

**Table 1:** Summary of passive and active nanoparticle separation microfluidic techniques.

	Techniques	Principles for separation	Applicable nanoparticles	Advantages	Limitations	Refs.
Passive techniques	DLD	Size	EVs, biomacromolecule, polystyrene particles	Simple operation; High resolution	Low throughput; Channel clogging; Requires specialized equipment and experience	[104, 105, 106]
	Inertial microfluidics	Size	EVs, polymersomes	High throughput; Simple operation; Good biocompatible	Low separation resolution; Processing capacity for nanoscale particles is limited	[139, 140]
	MF	Size	EVs, exosomes, biomacromolecule, nanoparticles	High separation efficiency; Easy to operate	Membrane clogging; Potential damage to soft particles due to shear stress	[108, 143, 144, 145]
	PFF	Size	EVs, virus, polymersomes	Simple to design and manufacture	Low throughput and resolution	[31, 143, 149]
	Viscoelastic microfluidics	Size	EVs, polymersomes	Higher separation resolution; Simple operation; Good biocompatible	Low throughput; Potential contamination	[37, 116, 156, 157]
Active techniques	Acoustofluidics	Size	EVs, exosomes, viruses biomacromolecule, polymersomes	Biocompatibility; Versatility; High resolution	Potential damage to nanoparticles due to heat generated by high frequencies	[173, 177, 178]
	MCE	Size; Charge; Polarity	EVs, exosomes, viruses, AuNP, polymersomes	High separation efficiency and resolution; Highly controllable; High throughput	Potential damage to biological particles by electric field contact and heat production	[192, 196, 197]
	FFF	Depends on the mediated field	EVs, exosomes, AuNP, polymersomes, biomacromolecule	High efficiency; Biofriendly; The appropriate different force fields can be applied	The equipment and operation are complex; Need long time separation	[152, 211, 219]
	Magnetophoresis	Magnetic susceptibility; Size	Paramagnetic, diamagnetic particles, ferromagnetic particle	Strong effect and specificity on magnetic particles; Fast response, high throughput	Limited scope	[31, 220, 221]
	Optofluidics	Size, Optical properties	Metal particles, polymersomes, bacteria	Highly flexible and adjustable; No label; Wide range of maneuverability	Potential damage of light and heat to particles; High equipment requirements; Depends on the optical properties of the particle.	[234, 239, 241]

#### 3.4.1 Hybrid Microfluidic Techniques Based on Immunoaffinity

Immunoaffinity technology, known for its high specificity, has been extensively applied in various applications for isolation, screening, and detection.^[^ ^43^, ^244^, ^245^, ^246^ ^]^ Unique among separation technologies due to its labelled nature, immunoaffinity offers exceptional specificity and accuracy. However, it also comes with higher costs, a limited application range, and requires specific expertise and insights for certain post‐processing operations.^[^ ^247^, ^248^, ^249^ ^]^ Since the other techniques described above do not directly interact the ligands or antibodies used in immunoaffinity, hybridization with other techniques is generally achieved by using a carrier, such as an immunomagnetic bead, or by modifying the antibody to attach it to a particle or substrate for separation and capture purposes.

**Figure 13:**
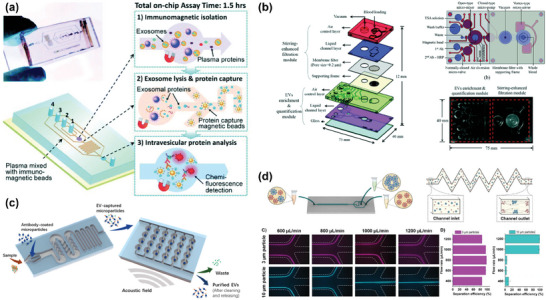
Hybrid microfluidic techniques based on immunoaffinity. a) Image of the prototype PDMS chip containing a cascading microchannel network for multi‐stage exosome analysis. The workflow for on‐chip immunomagnetic separation, chemical lysis, and intravesicular protein analysis of circulating exosomes. Reproduced with permission.^[^ ^250^ ^]^ Copyright 2014, Royal Society of Chemistry. b) Integrated microfluidic chip consists of 9 steps, a stirring‐enhanced filtration module, an EVs enrichment module, and an EV quantification module. Reproduced with permission.^[^ ^251^ ^]^ Copyright 2019, Royal Society of Chemistry. c) The sample and the antibody‐coated particles are completely mixed so that the particles capture the EV, and then the particles are enriched using acoustic waves. Reproduced with permission.^[^ ^252^ ^]^ Copyright 2016, Multidisciplinary Digital Publishing Institute. d) After the immune microbeads capture the target sEV, the small particles are concentrated in the channel side of the inertial fluid, while the large particles are concentrated in the channel center, so as to carry out size‐dependent separation. Reproduced with permission.^[^ ^253^ ^]^ Copyright 2023, Elsevier.

Immuno‐magnetic beads represent a classic hybrid approach, adept at overcoming challenges associated with nanoscale particle manipulation. For example, Mei et al. enriched and captured common exosome and tumor‐specific markers by immunomagnetic methods and demonstrated the phenotype of exosome subsets by performing multiparameter analysis of intravesical biomarkers in selected subsets (**Figure** **13**a). They selectively isolated exosomes from non‐small cell lung cancer plasma and quantified the total expression and phosphorylation of insulin growth factor receptor type 1 (IGF‐1R), compared with conventional methods. This method can directly separate subpopulations and quantitatively detect surface and intracystic biomarkers from minimally invasive plasma samples (30 µL) within ≈100 min, and the detection sensitivity is significantly improved, which is conducive to improving the accurate screening of cancer and non‐cancer diseases.^[^ ^250^ ^]^ Chen et al. built a highly integrated module for enriching and quantifying circulating EVs in whole blood sample.^[^ ^251^ ^]^ This module sequentially employs microfluidic filtration, an EV enrichment device using immunomagnetic beads, and an enzyme‐linked immunosorbent assay (Figure 13b). The vesicle recovery rate reached 94%, though the isolated EVs were not utilized further. Liu et al. combined acoustic filed with immunoaffinity to specifically capture and manipulate biological EVs, in which Her2‐positive EVs were successfully isolated and purified for further downstream analysis (Figure 13c). This technology opens up new possibilities for precise diagnosis and prognosis of EVs‐based diseases, such as cancer. In addition, this technique can be widely used to isolate and purify other biological targets, such as viruses and biomolecules. However, as mentioned in Equation (12), the acoustic mobility exerted on nanoparticles is usually weak, which may limit its processing capacity.^[^ ^252^ ^]^ Additionally, Bazaz et al. merged immunoaffinity with passive techniques like Immuno‐Inertial microfluidics, in which cancer cell‐derived small EVs (sEVs) were first captured on microbeads of different sizes, functionalized with specific trapping antibodies (Figure 13d).^[^ ^253^ ^]^ These microbeads were then introduced into a series of inertial microfluidic channels. With microbeads of different sizes bearing specific antibodies, various sEVs subpopulations were separated based on size, achieving over 90% separation efficiency. These isolated cancer‐specific small EVs can be further analyzed using flow cytometry, genomic and proteomic analysis, providing a foundation for clinical precision cancer characterization and offering enhanced insights into cancer management and monitoring.

The integration of immunoaffinity with other microfluidic technologies presents a significant advantage in simplifying nanoparticle manipulation. In these hybrid strategies, biological particles with varying surface markers are differentiated by immune microbeads. The larger size of these microbeads simplifies particle manipulation and enhances processing throughput. However, common challenges include costly labelling procedures, labor‐intensive preparation and washing steps, and the potential for captured molecules to contaminate the sample or alter the properties of the biological particles.

#### 3.4.2 Hybridization of Different Passive Microfluidic Techniques

Hybridization of passive microfluidic techniques is usually implemented through serial integration. For example, Liu et al. connected modular MFs in series to achieve cascade fractionation of EVs and exosomes.^[^ ^144^ ^]^ Kim et al. constructed two DLD devices in opposite directions to achieve focusing and separation functions.^[^ ^106^ ^]^ However, the hybridization of different non‐external field mediated microfluidic techniques does not show much superiority, as most of these methods mainly reply on particle size for separation.^[^ ^36^, ^254^, ^255^ ^]^

**Figure 14:**
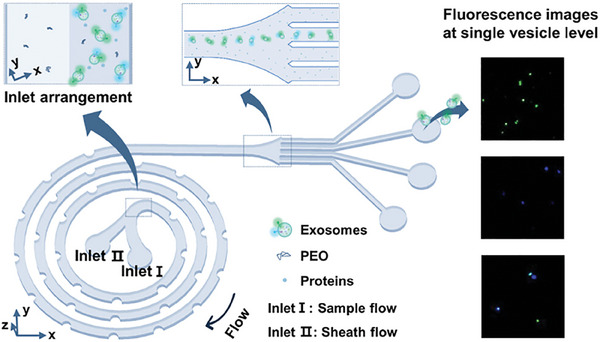
Dean‐Flow‐Coupled Elasto Inertial strategy for exosomes field‐free focusing/separation in the presence of protein contaminants. The viscoelasticity of the fluid is achieved by adding 0.15% of poly‐(oxyethylene) (PEO), resulting in single streak focusing of nanoscale particles under the leverage of elasticity, inertia, and Dean flow effects. Among them, Dean secondary flow generated by the concave structure in the spiral channel is conducive to particle focusing, which further promotes the sorting of nanoscale particles. Reproduced with permission.^[^ ^256^ ^]^ Copyright 2023, American Chemical Society.

Although hybridization strategies may not always enhance separation accuracy, they are able to reduce sample waste and allow for faster processing. Bai et al. exemplified this with their development of a Dean‐Flow‐Coupled Elasto‐Inertial strategy (**Figure** **14**a).^[^ ^256^ ^]^ By using a spiral microchannel structure, they created greater first normal stress differences, enhancing the viscoelastic effect for particle focusing. Additionally, concave structures within the channel generated Dean flow, further accelerating particle focusing. This approach enabled straightforward, field‐free purification and achieved high recovery of exosomes from as little as 50 µL samples. As a proof of concept, this technology showed the potential of combining analysis of individual vesicle‐level biomarkers (EpCAM and PD‐L1) with simultaneous aptamer‐mediated analysis for clinical precision diagnosis.

#### 3.4.3 Hybridization of Different Active Microfluidic Techniques

**Figure 15:**
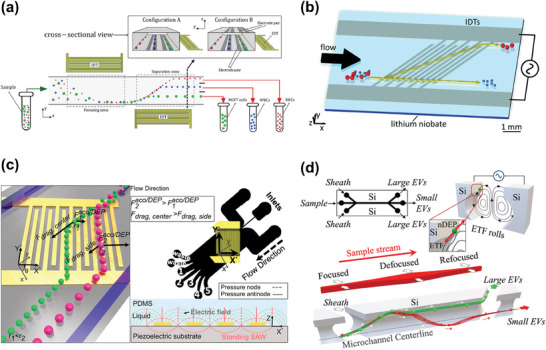
Hybridization between active microfluidic techniques. a) Combination of a focusing module and a sorting module, the focusing module focused all particles through TSAW, and then amplified the particle size difference through the joint action of the forces of TSAW and DEP, achieving biofriendly particle sorting with lower energy transfer and higher throughput. Reproduced with permission.^[^ ^260^ ^]^ Copyright 2021, Elsevier. b) an array of IDT on a piezoelectric lithium niobate substrate, and DEP was generated on both sides of the fluid. Reproduced with permission.^[^ ^261^ ^]^ Copyright 2014, Royal Society of Chemistry. c) Working principle and improved vDLD device structure. Reproduced with permission.^[^ ^262^ ^]^ Copyright 2019, American Chemical Society. d) The electrothermal fluid rolls made the particles roll horizontally in the microfluidic channel, and the small particles escaped the nDEP force and returned to the center of the flow line, while the large particles were captured by the nDEP force, thus realizing particle separation. Reproduced with permission.^[^ ^263^ ^]^ Copyright 2023, Royal Society of Chemistry.

The most commonly reported methods of hybridization of active microfluidic techniques are the combination of acoustic and electric fields or dielectrophoresis. Acoustic field affects the motion of particles over long distances, while electric field influences the motion of particles near the electrode. The simultaneous occurrence of these two fields does not interfere each other, but instead creates a continuous force that amplifies the small differences in the motion of particles. Although these techniques have been reported for the separation of various particles, their applications in nanoscale or high‐performance high‐resolution particle separation operations are lacking, which require higher energy mediations and may lead to temperature increases, cavitation, and electrolysis.^[^ ^31^, ^167^, ^257^ ^]^ For example, Ayala‐Mar et al. proposed a direct current–insulator‐based dielectrophoretic (DC‐IDEP) device capable of recovering exosomes at 75 and 120 nm at a voltage of 2000 V.^[^ ^258^ ^]^ However, such a high operating voltage is likely to cause rapid electrothermal flow and Joule heat, which detriments the continuous operation of the separation process and particle biology.^[^ ^259^ ^]^ Derakhshan et al. achieved a faster processing throughput of 20 µL min^−1^ while requiring a much lower voltage of 20 V (**Figure** **15**a).^[^ ^260^ ^]^ This significant reduction in voltage minimizes potential damage to biological particles, a notable advancement from the combination of technologies. However, this strategy is not effective for nanoparticles.

To address this limitation, Collins et al. combined acoustic fields with DEP (Figure 15b).^[^ ^261^ ^]^ This integration formed a DLD‐like separation structure (vDLD), with a set of IDTs in the microfluidic chamber generating force fields angled relative to the flow direction. The critical diameter determined by viscous resistance and the composite force field causes larger particles to move laterally along the minimum force potential line, while smaller particles follow fluid direction, enabling the separation of particles down to 300 and 500 nm. This team further optimized the structure (Figure 15c), and developed a tilted IDT structure, enabling particles to be influenced simultaneously by fluid resistance, acoustic radiation force and DEP force field. This structure facilitates lateral translation and, for the first time allows for the active separation of EVs (> 300 nm) and exosomes (< 200 nm). The performance was significantly improved, with purity exceeding 95%, meeting the gold standard method for exosomes, and the recovery rate being ≈1.4 times higher than that of the vDLD method (> 81%).^[^ ^262^ ^]^ The combination of ARF/DEP fields enables active sorting of EVs with high purity of size‐specific subsets, and the high‐quality purification makes EVs ideal disease biomarkers and drug delivery vectors.

In addition, some other field‐field combinations also provide better resolution and accuracy. For example, Bu et al. realized label‐free size fractionation of EVs by combination of electrothermal fluid (ETF) rolls and DEP in a microfluidic device (Figure 15d).^[^ ^263^ ^]^ In this system, EVs experienced negative DEP (nDEP) in a conductive medium, such as physiological or biological solutions. The DEP force diminishes rapidly with increasing distance from the field source (refer Equations (16)–(19)). To exploit this, non‐uniform Joule heating is employed to generate ETF rolls in the medium, leading to gradients in dielectric constant and conductivity. When the larger EVs are constantly transferred to both sides of the channel, they are subjected to the action of nDEP and do not return to the center of the flow line. Thus, the separation of EVs of different sizes was realized. By adjusting the frequency, peak voltage, and channel distance to cooperatively modify the capture range of the outer cycle and DEP forces, multiple separation objectives can be achieved. This strategy demonstrates acceptable separation capability down to particle size at ≈200 nm.

The tunability and synergy between different active fields allow their hybridization to overcome the difficulties associated with high‐precision manufacturing for separating smaller sizes and resolutions. However, the complexity of the sample particles themselves needs careful consideration, and the compatibility between different technical characteristics should be balanced.

#### 3.4.4 Hybridization Between Passive and Active Microfluidic Techniques

A diverse range of hybridization techniques combines passive and active microfluidic techniques, including DLD combined with DEP/electric fields,^[^ ^104^, ^121^, ^122^, ^206^, ^264^, ^265^ ^]^ inertial microfluidics‐DEP/Electric/Acoustic/Magnetic fields,^[^ ^266^, ^267^, ^268^, ^269^ ^]^ viscoelastic microfluidics‐electric /magnetic fields;^[^ ^270^, ^271^ ^]^ PFF‐Optical/Electric fields.^[^ ^272^, ^273^ ^]^ Among them, hybridization techniques based on DLD have great potential for applications in nanoparticle separation.

Stolovitzky et al. demonstrated the feasibility of DLD for nanoparticle manipulation with excellent resolution (down to 20 nm).^[^ ^106^, ^118^, ^123^ ^]^ However, it demands extremely high‐precision device fabrication techniques and equipment. Even the most efficient optimized design requires nanoscale spacing between pillars, which is the limitation of DLD technique itself. It also has shortcomings including low throughput, risk of blockage. Moreover, the high pressure induced by the supporting flow rate can cause irreversible damage to nanoparticles.

**Figure 16:**
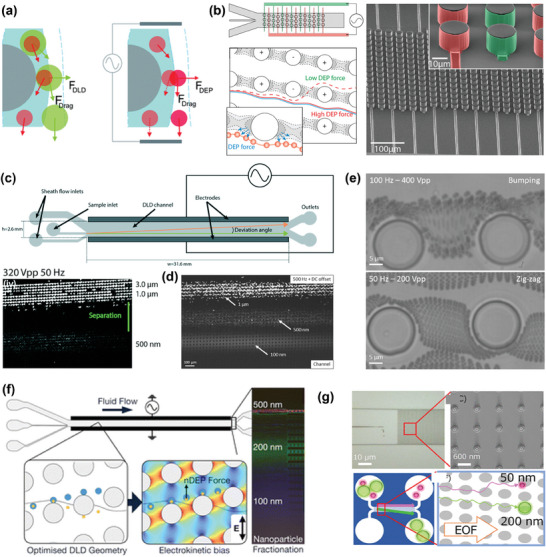
Representative examples of Hybridization between DLD and DEP/Electric field. a) Taking the force generated by DEP as an example, the field‐based strategy of hybridization with DLD reduced the *D_c_* without changing the DLD parameters by applying an additional force to the particle to help it enter the next streamline trajectory. Reproduced with permission.^[^ ^121^ ^]^ Copyright 2009, Royal Society of Chemistry. b) Alternating positive and negative electrodes were established on the DLD pillar to create an electric field gradient, which resulted in high and low DEP forces acting on the particles in the system. By adjusting the voltage, the ground critical diameter of the DLD could be reversibly reduced from 6 µm to 250 nm. In the DLD structure, G = 11.10 ± 0.14 µm. Adapted with permission.^[^ ^264^ ^]^ Copyright 2019, John Wiley & Sons. c) Based on DLD structure in d), AC electric fields were introduced to further narrow down *D_c_*. Reproduced with permission.^[^ ^275^ ^]^ Copyright 2019, AIP Publishing. e) At the same oscillation amplitude, 100 Hz and 400 Vpp signal makes particle bumping on the posts; 50 Hz and 400 Vpp signal makes particle zigzagging around the posts. Reproduced with permission.^[^ ^277^ ^]^ Copyright 2020, Elsevier. f) An alternating electric field was applied orthogonally in the direction of fluid flow to reduce the intrinsic critical diameter of DLD by ≈10 times. Reproduced with permission.^[^ ^274^ ^]^ Copyright 2022, Royal Society of Chemistry. g) EO drived DLD to realize nanovesicle separation. Nanoscale chip manufacturing technology and equipment were needed. Reproduced with permission.^[^ ^278^ ^]^ Copyright 2019, American Chemical Society.

Tegenfeldt et al. has attempted to incorporate DEP into DLD to reduce the fabrication requirement for separation.^[^ ^121^ ^]^ The additional force provided by DEP allows smaller particles that would otherwise flow along the original flow line to flow into the next flow line, thereby reducing the *D_c_* without changing the parameters of the DLD (**Figure** **16**a). After verifying the strategy using polystyrene polymer fluorescent microspheres with diameters of 2, 3, 5, and 10 µm, they used electrically connected metal‐coated pillars and applied an electric field in a DLD device (Figure 16b). The electric field gradient caused by alternating positive and negative electric fields of adjacent pillars can produce DEP forces on the polarizable particles that depend on their volume and polarizability, thus pushing particles close to or smaller than *D_c_* into the next streamline trajectory.^[^ ^264^ ^]^ Comparably, the *D_c_* of DLD in the hybridization strategy by Stolovitzky et al. was ≈6 µm.^[^ ^106^, ^123^ ^]^ With the adjustment of electric field intensity and frequency, the *D_c_* could be reduced to 250 nm at most (a 24‐fold reduction), and particles with a diameter below 100 nm could be successfully separated with acceptable DLD manufacturing requirements.

Morgan et al. also conducted a series of studies to introduce electric fields into DLD technique.^[^ ^274^, ^275^, ^276^, ^277^ ^]^ Initially, they introduced alternating current (AC) by integrating planar electrodes into a DLD device (Figure 16c) and found that high – and low‐frequency AC electricity caused the particles to behave differently at 3 µm, 1 µm, and 500 nm particles.^[^ ^276^ ^]^ At low frequencies (up to 500 Hz), the particles oscillate along the direction of the field under the influence of electrophoretic (EP)/electroosmotic (EO) forces. When the frequency of the magnetic field increases, the amplitudes of these oscillations disappear, and eventually the DEP generated by the inhomogeneous electric field becomes the main additional force affecting the particle behaviour. Interestingly, both the mechanisms alter the path of particles inside the DLD device, thereby enhancing the sorting of particles smaller than the critical diameter of the device. To this end, they quantitatively studied the differences between the two frequency ranges in depth,^[^ ^277^ ^]^ discovered that the particle deviation at low frequencies is proportional to the square of the size of the electric field, rather than the induced deviation thought by the previous studies to be caused by electrophoresis, where the amplitude of the oscillation is proportional to the size of the magnetic field. As shown in Figure 16e, at the same oscillation amplitude, 100 Hz/400 Vpp made the particles bump on the posts, while the condition of 50 Hz/200 Vpp makes the particles zig‐zag around them, indicating that the influence of electric field makes the particles behave differently, rather than EP oscillation. Meanwhile, they also combined DC with AC,^[^ ^275^ ^]^ further increasing the tunability and capability of particle separation (Figure 16c). The orthogonal application of DC and AC electric fields integrated to DLD allowed particle size and zeta electricity to be used simultaneously. The DC voltage passes through Faradaic processes at the electrode to change the conductivity of the local medium and form an electric field gradient, orthogonally formed nonuniform electrophoretic, contributing to narrowing down of *D_c_* thus the successful separation of 100, 500 and 1000 nm particles (Figure 16d). However, the introduction of electric fields usually causes potential harm to biological particles, thus They therefore explored its usability in the separation of soft vesicles (400 and 690 nm) with this strategy and found that the *D_c_* needed to be increased to ≈1 µm to guarantee extruded vesicles integrity (Figure 16f).^[^ ^274^ ^]^ In addition, Hattori et al. used EO‐driven DLD to achieve more accurate dimensional separation, but this came at the expense of increased difficulty in manufacturing DLD devices (Figure 16g).^[^ ^278^ ^]^

### 3.5 Comparative Analysis of Different Separation Methods

For potential users, the most concerned issue is to select the specific microfluidic separation technology to meet the needs. To this end, it is important to evaluate and compare the cutting‐edge technologies, including size‐based separation ability, resolution, required fluid properties, processing capacity, and fabrication difficulty (availability). **Table** **2**. listed the priority performances (based on size) reported for various techniques in nanoparticle separation till now. It includes details about their materials and manufacturing processes, as well as the types of nanoparticles successfully separated. It's important to note that results with experimental validation were listed here, which excludes those based solely on theoretical calculations.

**Table 2:** The priority performances (based on size) reported of techniques in nanoparticle separation till now.

Techniques		Separation capacity	Processing throughput	Particle	Materials of chip	Manufacturing technology	Publication year	Refs.
Passive techniques	DLD	20 nm	0.1–0.2 nL min^−1^	PS beads; Exosomes	200 mm wafer	Lithography and etch processes	2016	[123]
	Inertial microfluidics	200 nm	100–1400 nL min^−1^	Green, fluorescent polystyrene microsphere	SU‐8 patterned silicon wafer	Photolithography	2017	[142]
	MF	30 nm	3.3 µL/h–40 mL h^−1^	EVs	PMMA plates; track‐etched polycarbonate and polyethersulfone (PES) filter	Nut connection	2017	[144]
	PFF	30 nm	200 µL min^−1^	PS particles; EVs	PDMS	Lithography	2017	[152]
	Viscoelastic microfluidics	100 nm	200–3000 µL h^−1^	PS particles; EVs	SU‐8 patterned silicon wafer	Standard soft lithography	2023	[160]
Active techniques	Acoustofluidics	150 nm	0.2–0.6 µl min^−1^	PS particles; EVs	Silicon dioxide and aluminum nitride	–	2022	[169]
	MCE	130 nm	150 µL h^−1^	EVs	Bond	Polycarbonate sheets	2018	[197]
	FFF	5 nm	0.5–2 mL min^−1^	AuNPs	–	–	2011	[216]
	Magnetophoresis	200 nm	60 µL h^−1^	PS particles; EVs	PDMS	Standard soft lithography	2020	[228]
	Optofluidics	50 nm	100–300 particles min^−1^	AuNPs	SU‐8 patterned silicon wafer	Standard soft lithography	2016	[237]
Hybrid techniques	Inertial‐Viscoelastic microfluidics	200 nm	–	PS particles; EVs	PDMS	–	2023	[256]
	DEP‐Acoustofluidics	200 nm	0.75–3 µL min^−1^	PS particles; EVs	SU‐8 patterned silicon wafer	Standard soft lithography	2021	[262]
	DEP‐ETF	≈200 nm	0.04–0.06 mL h^−1^	PS particles; EVs	Silicon wafer; PDMS	–	2023	[263]
	DEP‐DLD	100 nm	–	Microspheres	SU‐8 patterned silicon wafer	Standard soft lithography; Sputtering	2019	[264]
	DLD‐Electronic field	100 nm	–	PS particles; EVs	SU‐8 patterned silicon wafer	Standard photolithography	2022	[274]

“‐” represents not mentioned in the corresponding reference.

In general, passive techniques are generally friendly to bioactive particles and do not easily cause soft particle damage, but the separation is only mediated by the size‐differential nature. The active techniques make more use of other properties of particles (such as shape, charge, magnetism, optical properties, etc.) for separation, so it can meet more diverse separation purposes and reduce the fabrication difficulty of the chip itself. However, it cannot be ignored that with the increase of particle manipulation difficulty, the increase of energy required will further increase the potential damage to soft or bioactive particles. Hybrid techniques can further take advantage of different particle properties, such as combining DEP and DLD technology, to achieve better separation effect and mitigate potential particle damage by simultaneously using particle size and their polarization characteristics. In addition, fluid properties can significantly affect particle behavior, most notably inertial microfluidics and viscoelastic microfluidics. As mentioned in the corresponding section of this paper, since inertial microfluidics usually maintain *Re* in the range of 1–100 (the *Re* of viscoelastic fluids is much less than 1), which makes the inertial term becomes more important than the viscous term, thus mediating the different motions of the particles. Therefore, especially when using undiluted biological liquids, they generally follow the action mode of viscoelastic microfluidics.

## 4 Various Applications of Microfluidic Nanoparticle Separation Methods for Precision Medicine

Microfluidic‐based nanoparticle separation plays a crucial role not only in rapid purification of biomarker particles for more accurate diagnostics, but also in the production of high‐quality nanomedicines for vaccine or therapeutic applications. The COVID‐19 pandemic underscored an urgent need for rapid and sensitive diagnostic, and monitoring approaches, as well as rapid vaccine manufacturing strategies. In the era of precision medicine or personalized medicine, where manufacturing small dosages for individual patient, such as personalized cancer vaccine becomes the norm, microfluidic platforms have emerged as valuable tools.^[^ ^33^, ^91^, ^279^ ^]^

### 4.1 Liquid Biopsy

Tissue biopsy, as a traditional method of detection, is not well‐suited to the needs of precision medicine. For instance, accurately determining and sampling biopsy sites requires elaborate screening and the expertise of medical professionals. However, in clinical practice, it is challenging to determine disease site and access lesions located in deep or perilous regions, which can lead to an underestimation of disease heterogeneity.^[^ ^280^, ^281^ ^]^ In addition, the sampling of biopsy is an invasive procedure. In contrast, liquid biopsy, as a non‐invasive technique, is able to analyze various readily accessible biological fluids (such as blood, urine, saliva, and cerebrospinal fluid), thus holding the potential for dynamic monitoring of diseases. Additionally, the analysis of body fluids can provide a systemic overview of changes without the need for specific lesion localization.^[^ ^7^, ^282^, ^283^ ^]^ Indeed, the various biological particles carrying lesion information within the bloodstream, including secretory proteins, and EVs, can provide clinicians with a sensitive and sustainable insight into the dynamic evolution of patients.^[^ ^284^ ^]^ Therefore, it is critical to meticulously separate and enrich these complex mixtures of biological samples on a small scale.

Traditionally, liquid biopsy relied on traditional centrifugation and filtration methods to complete the pre‐analysis phase of standardization, which often required larger volumes of biological fluids, resulting in limited detection and analysis of biomarkers due to the inefficiency of these techniques.^[^ ^285^ ^]^ The incorporation of microfluidic technology has revolutionized particle separation techniques, bringing substantial improvements in precision, efficiency, and throughput for biomarker analysis. Microfluidic separation has been transformative, offering unprecedented sensitivity and purity in isolating rare biological particles. This leap in capability highlights the critical advancements microfluidic technology has brought to the forefront of liquid biopsy, showing great potential in Point‐of‐Care Testing (POCT).^[^ ^280^, ^284^ ^]^

#### 4.1.1 Proteins and Peptides

The concept of utilizing proteins and peptides in bodily fluids as biomarkers dates back to the 19th century. During that period, the presence of proteins in urine had already been recognized as an important indicator for kidney diseases.^[^ ^286^ ^]^ Subsequently, the evolution of biochemical techniques has facilitated the analysis of a more diverse array of protein biomarkers in blood, which are now integral to clinical diagnostics.^[^ ^287^ ^]^ These biomarkers have become essential in the assessment of diseases, from early detection to prognosis of clinical practice. Over time, a wealth of data has accumulated, leading to the establishment of standardized evaluation criteria for numerous indicators. Compared to other biomarkers, proteins can most intuitively represent internal body processes, and their high specificity facilitates detection and separation. However, their complexity in bodily fluids and instability *ex vivo* poses challenges for conventional methods, especially under the context of precision medicine.

Although molecular weight differences are a key distinguishing feature of proteins or peptides, considering the complex 3D structures of proteins and their relatively small particle sizes (typically below 10 nm), solely size‐based separation methods become less applicable in this context.^[^ ^288^, ^289^ ^]^ In microfluidic separation and enrichment, their charge, affinity, shape, isoelectric point, and hydrophobicity are considered more comprehensively. Despite downsizing traditional chromatography techniques (e.g., gel filtration, ion exchange chromatography, and affinity chromatography) and electrophoresis onto a chip,^[^ ^290^ ^]^ FFF was also applied to isolate proteins and their complexes.^[^ ^291^ ^]^ Due to the strong affinity and specificity of proteins, most studies focus on the isolation and enrichment of proteins from complex biological samples. For example, Lin et al. developed an automated microfluidic chip for the rapid purification of target peptides in saliva samples.^[^ ^292^ ^]^ This chip utilized five connected pneumatic micromixers to automate the separation of target peptides. Under optimal operating conditions, the peptide recovery rate of the micromixer within 30 min was comparable to that of the traditional KingFisher system over a standard 2 h operation time. Hu et al. developed an efficient microfluidic system for extracting proteins with low molecular weight from biological fluids.^[^ ^293^ ^]^ By combining a polydimethylsiloxane layer with a nanoporous silica substrate, their system used the size‐exclusion characteristics of silica nanopores to selectively remove proteins of high molecular weight while enriching proteins of low molecular weight. Saucedo‐Espinosa et al. demonstrated a method for the separation and enrichment of biomolecules within microdroplets.^[^ ^294^ ^]^ This method applied electric potentials through a polydimethylsiloxane‐carbon composite membrane interface to nanodroplets to achieve the separation and enrichment of biomolecules of different sizes and charges. They used electrophoretic forces to migrate biomolecules to one side of the droplets based on their net charge, then a Y‐junction split the droplets to generate offspring droplets enriched with biomolecules. They also showed how this system could be used to separate peptide fragments after digestion based on their charge differences in different offspring droplets. Sarkar et al. proposed a rapid, multiplexed, and economical affinity‐based separation method for proteins and cells using an inertial particle sorter device.^[^ ^295^ ^]^ This technique used different‐sized affinity‐capture microbeads in a spiral channel for high‐throughput separation. It efficiently processed milligram‐scale protein samples or millions of cells in minutes. It is also effective for isolating low‐abundance antibodies specific to various HIV antigens and rare HIV‐specific cells from blood.

Although assays based on affinity have been well established and widely used for the detection of proteins and peptides, the processing power of these methods for complex samples is still limited. For precision medicine, the research focus is shifting from the detection of a single biomarker to the comprehensive analysis of multiple biomarkers in complex body fluids,^[^ ^6^ ^]^ which brings the need for microfluidic technologies that can accurately and efficiently isolate specific proteins from body fluids.

#### 4.1.2 EVs

EVs appear to be an ideal body fluid biomarker for microfluid‐based biopsies in vitro for precision medicine. 1) All cells release EVs under normal physiological conditions or acquired abnormalities, so using these particles as biomarkers can be useful for comprehensive analysis of patients.^[^ ^296^ ^]^ 2) Their size, ranging from 30 to 2000 nm,^[^ ^57^ ^]^ exceeds that of common proteins and cfNA, making them well‐suited for size‐based label‐free separation methods, as previously discussed in this article. 3) They are able to stably exist in virtually all body fluids in vesicular structures, not limited to blood.^[^ ^297^ ^]^ 4) In blood, their abundance is significantly higher, particularly in cancer patients, where their concentration can be several times higher than in healthy individuals.^[^ ^298^ ^]^ 5) Their membranes present specific proteins such as CD9, CD63, CD81, TSG101, HSC70, etc.,^[^ ^57^ ^]^ making them equally suitable for affinity‐based capture and detection methods. 6) They encapsulate a wide array of biomolecules such as lipids, metabolites, proteins, and nucleic acids, providing a wealth of valuable information for precision medicine.^[^ ^296^, ^299^ ^]^ The conventional separation and enrichment methods mainly include centrifugation, ultrafiltration, and affinity capture, but they require large sample size, and can damage the integrity and biological activity of the sample, and potentially cause sample contamination.^[^ ^300^, ^301^ ^]^

For example, Hisey et al. developed a herringbone‐grooved microfluidic device to separate exosomes from serum samples by covalent functionalization with antibodies targeting general and cancer‐specific exosome biomarkers, CD9 and EpCAM. After capture, exosomes were released using buffer and neutralized to preserve stability. Downstream characterization via fluorescence microscopy, nanoparticle tracking analysis, flow cytometry, and scanning electron microscopy demonstrated efficient isolation while maintaining exosome integrity. Notably, western blot analysis revealed significantly stronger EpCAM bands in exosomes from sera of patients at later disease stages, highlighting its potential for cancer stage evaluation.^[^ ^302^ ^]^ In addition, technologies are being integrated with downstream detection methods for more accurate, rapid analysis. Park et al. developed a DEP‐ELISA‐based platform, which separated high‐purity sEVs from plasma using DEP, followed by a colorimetric sensor module. This platform showed three orders of magnitude higher sensitivity than traditional ELISA and achieved diagnostic accuracies of 94.2%, 98.6%, and 91.3% for breast, colon, and lung cancers, respectively.^[^ ^303^ ^]^ In vivo analysis is also advancing. Cong et al. used a microfluidic device to monitor PD‐L1‐positive sEVs in vivo by establishing extracorporeal circulation in mice. The separation process leveraged particle size differences and CD63 aptamer‐modified magnetic nanospheres for selective sEV capture. This method enables continuous, real‐time monitoring of PD‐L1 sEVs during tumor growth, facilitating early diagnosis, ongoing tumor monitoring, and accurate prognostic feedback.^[^ ^304^ ^]^

As mentioned above, DLD, viscoelastic microfluidic, FFF, acoustofluidics, magnetophoresis and other microfluidic‐based techniques have demonstrated their ability for EVs or exosomes label‐free separation. The hybridization of different techniques has further demonstrated the reduction of potential damage due to energy transfer and dense structures. Nevertheless, some current challenges should be considered. Complex samples, especially blood, should be set up with appropriate, effective pre‐processing steps to avoid clogging and non‐specific trapping. This can usually be solved by sequential connection of multiple functional modules. However, complex procedures inevitably increase losses and reduce reproducibility. In addition, current label‐free methods do not have good selectivity for different subtypes of exosomes with similar physical and chemical properties.

### 4.2 Therapeutic Nanoparticles

Liquid biopsy strives to accurately detect problems, while therapeutic nanoparticles (nanomedicine) focus on accurately solving problems by delivering drugs to target sites.^[^ ^14^, ^15^, ^19^ ^]^ Especially in the context of the rise of nucleic acid medicine, the diversity of nucleic acid functions and therapeutic combinations necessitates different requirements for nanoparticles,^[^ ^305^, ^306^, ^307^ ^]^ most notably as a variety of delivery systems, such as nucleic acid‐structured delivery carriers (DNA nanostructures, aptamer carriers, etc.),^[^ ^308^, ^309^ ^]^ organic compound‐based delivery carriers (liposomes, lipid nanoparticles, polymers, etc.),^[^ ^21^, ^22^, ^25^, ^74^, ^310^, ^311^ ^]^ inorganic nanoparticles (gold nanoparticles, silicon‐based nanoparticles, iron oxide nanoparticles, etc.),^[^ ^312^ ^]^ naturally derived nanoparticles (from animal/plant sources or endogenous exosomes, albumin, etc.),^[^ ^28^, ^313^ ^]^ and virus‐based carriers (virus‐like particles, adeno‐associated virus, retrovirus, lentivirus, etc.).^[^ ^314^, ^315^ ^]^

The growing understanding of nanoparticle properties is reshaping the production and clinical translation of nanomedicines. Key properties such as size, charge, and stiffness are crucial for in vivo behavior, especially in precision medicine. As discussed, clinical translation now requires not only the removal of impurities but also purposeful nanoparticle enrichment. Traditional methods, however, are size‐specific and have high loss rates, highlighting the need for microfluidic separation techniques. Fortunately, advances in materials science and 3D printing are reducing the cost of microfluidics, and the industry has optimized various parameters, making this technology more accessible and adaptable for diverse applications.

For industrial‐scale production, clinical translational aspects become more critical. Traditional therapies face challenges such as maintaining consistent properties, therapeutic activity, stability, and high manufacturing costs. Scaling personalized nanomedicines, like mRNA cancer vaccines or CAR T‐cell therapies, further complicates the process with additional issues in quality control and batch consistency.^[^ ^29^ ^]^ For instance, tissue‐derived sEVs (TDsEVs) more accurately reflect in vivo signals and exhibit better homing effects than in vitro derived sEVs, but scaling up production of patient‐derived samples is challenging.^[^ ^316^, ^317^, ^318^ ^]^ Therefore, industrial‐scale production in precision medicine focuses not on volume, but individualized dosage, aligning with microfluidic technology's capabilities.^[^ ^319^, ^320^, ^321^ ^]^ Microfluidics can easily scale up by connecting units in parallel, enabling efficient and reliable amplification once an effective microfluidic unit is established. In contrast, traditional amplification strategies require extensive troubleshooting, increasing time and labor costs.^[^ ^95^, ^248^ ^]^

The development of diverse manufacturing technologies is reducing the complexity and cost of microfluidic systems, making them ideal for the demands of personalized nanomedicine. By expanding processing capacity through parallelization, microfluidic separation improves production efficiency and reproducibility. As Langer et al. have noted, microfluidics bridges the gap between lab‐scale experimentation and GMP‐compliant production, ensuring the quality and integrity of precision medicine products.^[^ ^322^ ^]^

#### 4.2.1 Nanoparticle Purification for Continuous Production

Nanoparticles have been studied in benchtop research for decades, but their clinical translation and application remain limited, primarily due to challenges in the purification of nanomedicines.^[^ ^323^ ^]^ To address this, various purification and separation technologies have been successfully integrated into microfluidic chips as functional modules.^[^ ^324^ ^]^ This integration enables the direct production of delivery carriers that meet drug delivery requirements.^[^ ^41^ ^]^ For example, Xu et al. conducted kinetic analysis revealing that drug release from monodisperse particles occurred more slowly than from particles with the same average size but a broader size distribution, with a significantly lower initial burst. This suggests that particle size is crucial for drug release. Microfluidic flow‐focusing (FF) technology was employed to create homogeneous particle systems for drug delivery.^[^ ^325^ ^]^ Moreover, continuous processing on microfluidic chips reduces the risk of pathogen or impurity contamination and typically enhances processing speed. In the case of mRNA‐LNP, free mRNA has been shown to elicit unpredictable immune responses.^[^ ^326^, ^327^ ^]^ To address this, Gao et al. combined flow field‐flow fractionation with multi‐detectors to establish a continuous production platform for separating uncoated mRNA and mRNA‐LNP, offering insights into improving the efficiency and safety of therapeutic nanoparticles.^[^ ^328^ ^]^

**Figure 17:**
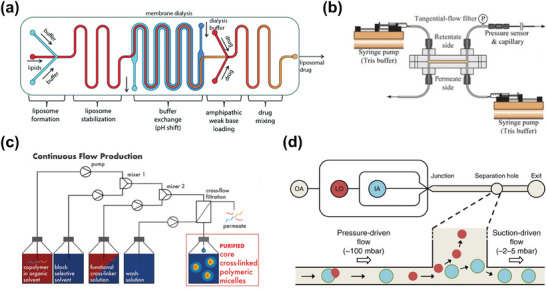
Purification for continuous production. a) Continuous flowing liposome/drug production system. liposome was first synthesized in the “liposome formation” region, then fully formed into a stable structure in the “liposome stabilization” section, purified in the “buffer exchange” region, and finally loaded with drugs. Reproduced with permission.^[^ ^329^ ^]^ Copyright 2014, Royal Society of Chemistry. b) The penetration/retention plates were clamped together to form a tangential flow filter unit. Reproduced with permission.^[^ ^331^ ^]^ Copyright 2012, Elsevier. c) TFF module was integrated into a continuous flow production strategy for the purification of delivery particles. Reproduced with permission.^[^ ^332^ ^]^ Copyright 2023, John Wiley & Sons. d) Double‐emulsion droplets formed at the OLA junction, which transform into unilamellar liposomes downstream as lipids assemble at the interface and 1‐octanol phases separate. The liposomes and 1‐octanol droplets are then directed into a separation hole, where droplets rise and liposomes are drawn into the post‐hole channel. Reproduced with permission.^[^ ^335^ ^]^ Copyright 2018, Springer Nature.

The concept of integrating manufacturing with isolation and purification into a microfluidic chip was proposed in 2014., Hood et al. pursued the concept of on‐site liposomal drug preparation, initially through a single‐step continuous flow microreactor.^[^ ^329^ ^]^ They achieved a high concentration of stable, loaded drug compounds and then implemented a membrane‐based countercurrent micro‐dialysis module. This module used large pH and concentration gradients for online buffer replacement (**Figure** **17**a). After loading and incubation of chemicals, purified liposomal drug preparations are generated in real time. This innovative approach not only minimized reagent waste but also drastically reduced the processing time from days to less than 3 min.

Similarly, Dimov et al. introduced a novel microfluidic process by employing TFF on a chip for continuous production and purification of liposomes.^[^ ^330^ ^]^ This method involved a replaceable membrane TFF device on the chip (based on their previous study,^[^ ^331^ ^]^ Figure 17b), capable of generating purified liposome products in under 4 min with non‐encapsulated drugs (Propofol > 95% reduction of unencapsulated drug) and or proteins (ovalbumin > 90% reduction of OVA), and organic solvents can be effectively removed (ethanol > 95% reduction). Bauer et al applied TFF for the continuous production of polymeric micelles (Figure 17c).^[^ ^332^ ^]^ The regenerated cellulose membrane removes residual polymers, crosslinkers, organic solvents, tri (2‐carboxyethyl) phosphine oxide, as well as DMSO and ethanol, thus allowing the process to be safely carried out without potentially hazardous components and toxic solvents. Recently, this strategy has also been applied to the online purification of proteins, rapidly removing protein denaturants and enabling high concentrations of active carbonic anhydrase to refold in just 20 min, a process several orders of magnitude faster than traditional dialysis methods,^[^ ^333^ ^]^ demonstrating its potential for applications in protein‐based carriers. Dekker et al. described a density‐based purification system to remove assembly reagent 1‐octanol from the fabricated cell‐sized liposomes and reach a purity of up to 95%.^[^ ^334^, ^335^ ^]^ As shown in Figure 17d, 1‐octanol droplets were finally separated in the form of droplets to form a monolayer liposome. The liposome and 1‐octanol droplets then entered the separation pore, and the droplets drifted upward while the liposome was slowly drawn into the post‐pore channel.

Obviously, TFF remains the most used technique for therapeutic nanoparticle purification in continuous production strategies based on microfluidics. However, this technique inevitably accompanies membrane clogging and potential damage to soft particles. As we discussed above, other purification techniques also have random factors that affect the final product quality. Given this, subsequent quality control (QC) is not only necessary but also an important aspect of complying with GMP.^[^ ^336^ ^]^

#### 4.2.2 Separation for Quality Control

In biopharmaceutical processing, purification steps usually occur between particles with significant size differences, such as mRNA and LNP. After purification, these particles still require further separation and characterization for quality control (QC). FFF stands out for its lack of stationary phase, allowing it to impose minimal shear and mechanical stress on the sample composition, which is particularly important for separation prior to quality control testing. At present, FFF on microfluidic has been successfully applied to the pre‐analytical separation procedures of nucleic acid,^[^ ^337^ ^]^ protein,^[^ ^338^ ^]^ exosome,^[^ ^339^ ^]^ LNP,^[^ ^340^ ^]^ virus‐like particles and other particles.^[^ ^341^ ^]^

However, few studies have incorporated this step as a separation strategy for delivery carriers in QC. In this context, it is suggested to consider the following points: 1) The size, uniformity, and concentration of the drug‐loaded particles must be measured to determine their viability; 2) The methods used should be suitable for current samples and not affect the morphology, encapsulation efficiency, or particle size of the already stabilized drug‐loaded particles; 3) The separation method must avoid introducing contaminants that could negatively impact the sample's stability or functionality; 4) The method should have sufficient resolution to differentiate between various particle populations, especially in multi‐component systems; 5) The separation process should be repeatable to ensure reliability and consistency across batches and laboratories; 6) The employed technology should allow for rapid, high‐throughput analysis to meet the pace of commercial production; 7) Efficacy consistency should be verified in multistage biological experiments.

## 5 Challenges and Perspectives

Microfluidic technologies have indeed demonstrated superior performance over traditional methods for separating bioactive nanoparticles. Yet, their application in clinical setting remains limited, with traditional separation methods still dominating. Beyond issues of reproducibility and sample adaptability, significant challenges arise from manufacturing techniques, equipment, and expertise. A common problem with these technologies is that as particle size decreases, separation becomes more difficult. This not only increases the risk of damaging biological and soft particles, but also reduces processing throughput. In addition, the challenge of fabricating effective passive techniques increase significantly as separation performance improves. For example, achieving high‐precision separation of 20 nm particles requires DLD structure accuracy of 25 nm, which needs expensive deep ultraviolet (DUV) lithography.^[^ ^123^ ^]^ For active techniques, although standard lithography remains a routine method for chip manufacturing, high‐precision chip structures are not essential. Consequently, advancements in 3D printing technology, particularly in light‐curing techniques and material science, have enabled it to partially replace traditional lithography, thereby improving accessibility.^[^ ^342^, ^343^ ^]^ With advancements in nanomedicine and the growing demand for personalized solutions, microfluidic separation technologies are expected to gradually transition from the lab to standard clinical applications. This review discusses the most promising technologies poised to address these challenges over the next 5–10 years, focusing particularly on the interdisciplinary potential offered by advancements in material science,^[^ ^342^, ^344^ ^]^ 3D printing technologies,^[^ ^345^, ^346^ ^]^ and data‐driven artificial intelligence.^[^ ^51^, ^52^, ^347^ ^]^

### 5.1 Manufacturing Challenge

Integrating microfluidics with nanomedicine presents significant manufacturing challenges. The high demands for specialized equipment and experience have limited the accessibility of microfluidics’ key attributes, such as customization, portability, and integration, to the broader public. However, advancements in 3D printing technology are driving rapid prototyping and personalized production of microfluidic chips, while developments in material science are enhancing chip performance and expanding their applications.

Typically, 3D models are designed using computer‐aided design (CAD) software and printed using a layer‐by‐layer process,^[^ ^345^ ^]^ allowing for flexible designs and rapid manufacturing. This enables fine‐tuning of 3D models based on empirical data to meet the specific requirements of personalized microfluidic structures. Currently, most microfluidic materials are based on polydimethylsiloxane (PDMS) using soft lithography technology, and it is not compatible with traditional 3D printing.^[^ ^342^ ^]^ Solutions has emerged, including indirect 3D printing approaches that create molds through 3D printing, into which PDMS is then poured or injected. Stereolithography and inkjet 3D printing use specialized print heads or photocuring systems to address PDMS's viscosity and curing challenges.^[^ ^344^ ^]^ Alternative materials, such as polystyrene and PMMA, offer improved thermoplastic properties and avoid issues associated with PDMS, such as the absorption of hydrophobic components.^[^ ^348^ ^]^ However, most current materials are chemically inert, and potential toxicity concerns have driven interest in developing customized materials.^[^ ^349^ ^]^ Moreover, achieving finer structures poses additional challenges, largely due to hardware limitations, which typically restrict resolution to 100 µm. Additionally, surface roughness from layer‐by‐layer manufacturing errors can impact the design of particle separation structures.^[^ ^350^ ^]^

To address the diverse requirements of particle handling and personalized needs, microfluidic device manufacturers should consider leveraging 3D printing technology. Establishing a reliable model database could offer guidelines and recommendations for various applications, creating a bridge from theoretical concepts to physical implementations and helping this technology move beyond a limited number of laboratories to broader, real‐world use.

### 5.2 Complex Biological Sample Handling

One of the main findings of this review is that few studies have explored the activities of biological particles post‐isolation, despite many techniques demonstrating nanoparticle manipulation. In addition, there is a scarcity of studies utilizing samples from human or animal origin. Reports that analyze whole blood often involve sample dilution before processing and employ series or parallel strategies within multifunctional modules to address sample complexity.^[^ ^160^, ^178^, ^251^, ^256^, ^265^ ^]^

Indeed, in addition to bodily fluid samples, there are also different types of body fluids, including lymph, cerebrospinal fluid, bone marrow, saliva, sweat, pleural effusion, cervical fluid, sputum, urine, and feces.^[^ ^351^ ^]^ Therefore, the first challenge is determining whether the separation process damages the activity of biomarker particles, as the loss of molecular markers and reduction of cell viability have been demonstrated.^[^ ^33^ ^]^ In addition, separation techniques in microfluidics are affected by fluid rheological properties, especially in precision medicine. For example, whole blood viscosity increases with cholesterol and triglyceride levels,^[^ ^352^ ^]^ implying that appropriate microfluidic parameters will vary between different patients, or even for the same patient at different stages of a disease.

When dealing with complex samples, although personalization is emphasized, standardization remains a necessary foundation. This involves the collection, processing, and downstream analysis of samples to control objective differences. Personalized measures can then be taken based on the characteristics of the patient's sample. Advancements in data‐driven 3D printing technology may offer a solution, as machine learning (ML) is accelerating the response speed of 3D printing designs to data feedback.^[^ ^353^ ^]^ Therefore, by establishing models for the separation of different samples, a manufacturing platform capable of rapid response can be developed. Another promising solution is to leverage the modular advantages of microfluidic technology. Currently, various combinations of microfluidic modules have been developed, allowing suppliers to offer customized combinations tailored to different sample requirements.^[^ ^95^ ^]^ Such modular approaches not only enhance the quality of fabrication but also ensure the repeatability and safety of the manufacturing process.

### 5.3 Artificial Intelligence: From Aid to Driven

The importance of data cannot be overemphasized, but without the aid of AI and ML, we would struggle to manage increasingly large and heterogeneous data sets. Benefiting from parallel advancements in algorithms and computing hardware, these technologies have demonstrated the ability to engineering designs^[^ ^51^ ^]^ and intelligently analyze complex detection data, such as genomics and proteomics.^[^ ^354^, ^355^ ^]^

In this review, we recognize the critical role of simulation in chip design, which requires balancing numerous parameters synergistically. However, after simulation, adjustments based on the actual conditions of specific particles in the real world are still necessary. Consider a specific scenario: if the properties of the fluid change with the progression of a patient's condition, can the existing system still be applied? Therefore, initial models based on first principles remain essential, but fine‐tuning for specific cases can be handed over to ML for rapid responses. For example, one study optimized the IDT function using ML based on acoustic fields, quickly identifying the optimal design parameters for efficient separation.^[^ ^356^ ^]^ The integration of AI with 3D printing technologies further enhances this capability, facilitating the rapid prototyping of microfluidic devices with complex geometries tailored to specific separation tasks.^[^ ^357^ ^]^

For specific case, the accuracy and stability of data are particularly important and directly affect the results of model output. However, when the detector is narrow down to the chip, the performance decline is generally inevitable, the most common case is the direct impact of ambient temperature and humidity on the spectrum.^[^ ^358^ ^]^ One promising solution is data‐fusion strategy‐driven AI that is capable of defining conditions from a high‐dimensional, multi‐scale perspective to capture the heterogeneity of complex diseases.^[^ ^347^ ^]^ As mentioned above, the body fluid biomarkers contain information from multiple sources. The separation system based on microfluidics can not only obtain more accurate detection results after separating them, but also obtain information of different biomarkers in parallel, which are different from each other but interrelated, and their internal complex relationships can be submitted to appropriate algorithms for analysis through fused data.

However, we often do not have sufficient data to train a robust model specifically for a particular task. Transfer learning could be a promising solution. In short, it is pre‐trained on large‐scale data and then fine‐tuned for a specific task. It has been applied across fields like material property prediction,^[^ ^359^ ^]^ drug design,^[^ ^360^ ^]^ manufacturing of liposomes,^[^ ^361^ ^]^ regionally selective and stereoselective reactions,^[^ ^362^ ^]^ medical image analysis,^[^ ^363^ ^]^ etc. Transfer learning also supports advancements in microfluidics, such as channels,^[^ ^364^ ^]^ personalized drug administration,^[^ ^365^ ^]^ and focused droplet generation.^[^ ^52^ ^]^ Additionally, physical laws governing microfluidic particle separation are now formalized into equations, providing a foundation for integration into Physics‐Informed Neural Networks (PINNs).^[^ ^366^ ^]^ By embedding these principles, PINNs offer a promising approach in data‐limited settings, enhancing the understanding and application of microfluidic separation.^[^ ^366^ ^]^ Therefore, this review highlights how these first principles form a basis for a pre‐trained model capable of yielding precise outputs with minimal task‐specific data, creating a versatile platform for high‐precision applications.

## 6 Conclusion

As precision medicine becomes more feasible, the role of microfluidic technologies in particle separation is emerging as a critical step in making this vision a reality. Current research and approaches may fall short in addressing the clinical challenges and opportunities presented by recent advances in treatment. Moreover, recent technological advancements are imposing higher technical demands. Microfluidics, based on the fundamental principles of separation, must now actively engage in interdisciplinary collaborations to fulfill promises of speed, personalization, and precision. Recent developments have demonstrated that on‐chip separation is complementing the field of nanomedicine, pushing boundaries in this area. For example, microfluidics has been increasingly utilized for effectively identifying and separating disease‐related biomarkers, crucial for early diagnosis and personalized treatment. On the other hand, the integration of microfluidic chips with machine learning and artificial intelligence is opening new avenues for data analysis and pattern recognition, thus significantly contributing to drug discovery and biomedical research. Additionally, the application of 3D printing technology and advanced materials is expanding the capabilities of microfluidic devices, allowing for more precise fluid control and facilitating more complex experimental designs. These advancements not only accelerate the development of new therapeutic methods but also enhance efficiency of disease monitoring and management. In summary, with ongoing technological progress and interdisciplinary integration, the potential of microfluidics in advancing precision medicine and personalized treatment is becoming increasingly evident.

## Conflict of Interest

The authors declare no conflict of interest.
